# On Bio-Inspired Strategies for Flow Control, Fluid–Structure Interaction, and Thermal Transport

**DOI:** 10.3390/biomimetics11020143

**Published:** 2026-02-13

**Authors:** Farid Ahmed, Leonardo P. Chamorro

**Affiliations:** 1 Department of Nuclear, Plasma & Radiological Engineering, University of Illinois Urbana–Champaign, Urbana, IL 61801, USA; farida2@illinois.edu; 2 Department of Mechanical Science and Engineering, University of Illinois Urbana–Champaign, Urbana, IL 61801, USA; 3 Department of Civil and Environmental Engineering, University of Illinois Urbana–Champaign, Urbana, IL 61801, USA; 4 Department of Aerospace Engineering, University of Illinois Urbana–Champaign, Urbana, IL 61801, USA; 5 Department of Earth Science & Environmental Change, University of Illinois Urbana–Champaign, Urbana, IL 61801, USA

**Keywords:** bio-inspired engineering, biomimetics, flow control, fluid–structure interaction, phase-change heat transfer, drag reduction, energy harvesting

## Abstract

Bio-inspired engineering draws on principles refined by natural evolution to tackle persistent challenges in fluid mechanics, structural dynamics, and thermal transport. This article presents a critical, mechanism-driven narrative review that integrates recent advances across three complementary domains that are often treated independently, namely: flow-control strategies such as leading-edge tubercles, alula-like devices, riblets, superhydrophobic skins, and hybrid low-Reynolds-number fliers; fluid-structure interactions inspired by aquatic and aerial organisms that leverage compliant foils, flexible filaments, ciliary arrays, and piezoelectric fluttering plates for propulsion, wake regulation, mixing, and energy harvesting; and phase-change heat-transfer surfaces modeled after stomata, porous biological networks, and textured cuticles that enhance nucleation control, liquid replenishment, and droplet or bubble removal. Rather than providing an exhaustive catalog of biological analogues, this review emphasizes the underlying physical mechanisms that link these domains and enable multifunctional performance. These developments reveal shared physical principles, including multiscale geometry, capillary- and vortex-mediated transport, and compliance-enabled flow tuning, which motivate the integrated treatment of aerodynamic, hydrodynamic, and thermal systems in applications spanning aerospace, energy conversion, and microscale thermal management. The review assesses persistent challenges associated with scaling biological architectures, ensuring long-term durability, and modeling tightly coupled fluid-thermal-structural interactions. By synthesizing insights across flow control, fluid-structure interaction, and phase-change heat transfer, this review provides a unifying conceptual framework that distinguishes it from prior domain-specific reviews. Emerging opportunities in hybrid multi-mechanism designs, data-driven optimization, multiscale modeling, and advanced fabrication are identified as promising pathways to accelerate the translation of biological strategies into robust, multifunctional thermal–fluid systems.

## 1. Introduction

Nature provides a rich source of optimized strategies for addressing engineering challenges involving fluid dynamics, structural response, and energy conversion. Bio-inspired engineering, including biomimetics, seeks to abstract operational principles from biological systems and translate them into engineered technologies, particularly in applications characterized by multiscale flows and compliant structures [1]. Successful translation requires careful consideration of scale mismatch, material constraints, and Reynolds-number regime differences between biological organisms and engineered devices [2,3]. Within fluid dynamics, bio-inspired approaches span morphology-based flow control and surface-driven transport manipulation, with the common objective of achieving robust performance improvements under practical engineering constraints [1,4].

Recent advances in bio-inspired fluid mechanics emphasize unsteady and transient flow phenomena that are difficult to realize using conventional engineered designs. In particular, studies of so-called survival hydrodynamics highlight how organisms exploit unsteady vortex formation, distributed flexibility, and rapid energy exchange with the surrounding fluid to achieve accelerations and maneuverability that exceed typical engineered capabilities [5]. These insights show the importance of unsteady flow physics and compliant structures as central mechanisms for bio-inspired performance enhancement, rather than historical or purely geometric analogies [6].

A significant area within bio-inspired engineering focuses on morphology-based flow control and the development of smart surfaces. This can be categorized into two primary archetypes [1]: (1) Devices attached to wings or lifting bodies designed to enhance overall aerodynamic or hydrodynamic performance, and (2) Engineered surfaces specifically developed for the purpose of skin-friction reduction. Examples of these bio-inspired designs span a wide range, from features like leading-edge bumps (inspired by humpback whale tubercles) to trailing-edge feathers, alula-like elements, and complex riblet or lotus-inspired textures. The successful implementation of these designs hinges on effectively reconciling biological kinematics and morphology with practical engineering considerations. Such considerations include managing the Reynolds-number mismatch between biological and engineered operating conditions, as well as ensuring manufacturability [1]. In addition to biological analogues, aircraft vertical tails and rudders represent long-standing engineering implementations of morphology-based flow control operating in regimes comparable to biological stabilizing appendages. Recent experimental studies at low freestream velocities and high sideslip angles demonstrate that vertical rudder geometry can delay separation, enhance control authority, and improve lateral stability through three-dimensional flow manipulation and vortex control [7].

At a fundamental level, many bio-inspired flow-control strategies can be viewed as responses to the onset and control of flow separation, which remains one of the dominant limiting factors in aerodynamic performance and convective heat transfer. Flow separation governs lift degradation, drag increase, thermal resistance, and unsteady loading across a wide range of engineering systems. A recent comprehensive review by Abdolahipour [8] systematically analyzed flow separation control using unsteady actuation, emphasizing the critical role of excitation frequency and momentum coefficient as the primary dimensionless parameters governing separation suppression and reattachment. Importantly, the frequency dependence identified in active flow control exhibits strong conceptual parallels with biological actuation. Low-frequency excitation can couple with large-scale vortex shedding, similar to wingbeat frequencies in birds or fin oscillations in fish, while high-frequency excitation alters near-wall shear-layer dynamics in a manner analogous to rapid micro-actuation observed in biological appendages. Recent developments such as modulated pulse jet vortex generators enable the simultaneous exploitation of low- and high-frequency actuation within a single control framework, offering enhanced separation control efficiency by combining wake-scale and shear-layer-scale mechanisms [8].

The lotus effect illustrates how hierarchical surface structures can regulate wettability and near-wall transport, enabling functionalities such as self-cleaning and passive drag reduction under suitable conditions [9]. Translating such biological surfaces into engineering applications requires preserving the underlying physical mechanisms rather than geometric similarity alone. These principles are directly relevant to thermal and transport systems, where controlled surface wettability and interfacial dynamics can be used to influence boundary-layer behavior, nucleation processes, and overall heat-transfer performance [10].

FSI provides the bridge from form to function. Haward and Shen [11] highlighted how FSI considerations span orders of magnitude in size and fluid rheology from vortex-induced vibration in large, high-Re infrastructures to viscoelastic microflows driven by cilia and flagella, where added relaxation times reshape stability and transport. This systems perspective is essential for designing bio-inspired components that perform reliably in real environments: flexible channel walls can sustain chaotic, mixing-enhanced states at unexpectedly low Reynolds numbers; viscoelastic wakes can be harnessed rather than avoided; and discrete symmetries of micro-objects can generate entirely new angular dynamics even in simple shear flows [11].

Data-driven methods, particularly deep reinforcement learning (DRL), have emerged as powerful tools for bio-inspired flow control and optimization by enabling the discovery of effective actuation and locomotion strategies beyond analytical design approaches [12]. Despite demonstrated successes, challenges related to sample efficiency, sim-to-real transferability, and computational cost continue to limit robust deployment in multiscale fluid systems [13]. In addition, bio-inspired optimization methods, including swarm intelligence and evolutionary algorithms, provide flexible frameworks for exploring multi-objective trade-offs in complex flow and heat-transfer design problems [14,15]. These methods complement physics-based modeling by efficiently navigating high-dimensional design spaces shaped by biological self-organization principles.

Despite prior reviews on biomimetics, bio-inspired flow control, and bio-inspired energy systems, the literature remains largely fragmented across application domains and physical processes, with limited synthesis at the level of governing transport mechanisms. Those typically emphasize either geometric inspiration, application-specific performance metrics, or isolated technologies (e.g., aerodynamic control, surface wettability, or energy harvesting), but rarely establish a unified fluid-mechanical framework that connects flow control, fluid–structure interaction, and phase-change transport under common scaling principles. As a result, key questions remain unresolved: which biological mechanisms yield fundamentally new transport pathways, which represent incremental optimization within classical scaling laws, and how performance and scalability are constrained by Reynolds number, compliance, multiphase effects, and energetic cost.

To bridge this gap, this review explores how bio-inspired designs can be systematically classified and evaluated according to their fundamental fluid-mechanical mechanisms, rather than their biological taxonomy or application domain. The scope is strictly delimited to fluid-driven systems within low-to-moderate Reynolds number regimes, where unsteadiness, structural compliance, and interfacial physics are the governing phenomena. Topics such as sensing, data-driven optimization, and artificial intelligence are discussed solely in their capacity to enable the discovery, control, or scaling of these primary mechanisms, rather than as independent subjects of inquiry.

The work is organized around three analytically connected thrusts: (i) bio-inspired flow control and drag or energy modulation, where morphology and surface physics alter momentum transport; (ii) bio-inspired fluid–structure interaction, where compliance and unsteady kinematics govern force generation and energy exchange; and (iii) bio-inspired phase-change heat transfer, where hierarchical surfaces regulate interfacial transport. Within each thrust, representative systems are compared using dimensionless parameters, normalized performance metrics, and explicit discussion of limitations, scale effects, and energetic trade-offs. This mechanism-driven organization distinguishes this review from prior surveys and provides an approach for identifying transferable design principles and prioritizing future research directions. Recent syntheses of advances in aerodynamics further emphasize that bio-inspired flow control, nature-inspired materials, and data-assisted modeling are central components of emerging aerodynamic research frameworks [16].

## 2. Roadmap and Scope of the Review

**Figure 1:**
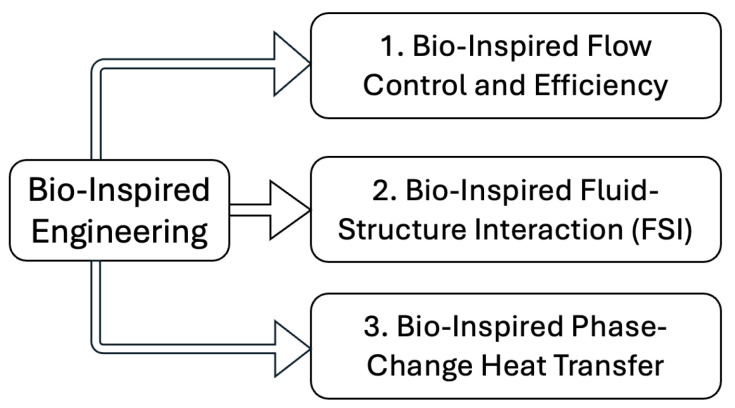
Roadmap illustrating the organization of the review and its three core domains in bio-inspired engineering.

The review is organized around three core domains of bio-inspired engineering that leverage nature’s strategies to enhance fluid performance. Figure 1 illustrates this structure: (1) bio-inspired flow control and energy-efficiency mechanisms that improve aerodynamic and hydrodynamic performance; (2) bio-inspired fluid–structure interaction systems that exploit flexibility and deformation for propulsion, mixing, and flow-energy conversion; and (3) bio-inspired phase-change heat-transfer surfaces that optimize nucleation, liquid resupply, and vapor removal. This approach reflects the growing coupling among surface architecture, structural compliance, and multiphysics interactions and guides the translation of biological principles into advanced thermal-fluid technologies.

## 3. On Bio-Inspired Advances in Fluid Mechanics

Progress in bio-inspired fluid mechanics has demonstrated how natural systems can guide the design of advanced engineering solutions. Particular efforts have gone into biological surfaces, structures, and locomotion strategies to improve drag reduction, propulsion, aerodynamic performance, and energy conversion. The driving motivation is that plants and animals have evolved efficient interactions with fluids through features such as textured skin, flexible appendages, and dynamic shape adaptation. These naturally optimized mechanisms directly inform new approaches to manipulate boundary layer behavior, stabilize separated flows, and convert mechanical energy more effectively.

### 3.1. Drag-Reduction Mechanisms from Marine Surfaces

Bio-inspired drag reduction technologies have emerged by learning from biological structures that manage turbulent flows efficiently in marine environments. Three main strategies have shown strong potential in reducing hydrodynamic resistance in underwater applications: non-smooth surfaces, superhydrophobic surfaces, and water-jet surfaces. Each strategy operates through a distinct mechanism and has reported significant improvements in fluid performance under specific conditions.

The riblet-inspired morphology from sharkskin denticles has been intensively studied due to its ability to influence turbulent structures at the solid-fluid boundary. Studies have demonstrated that riblets with optimized geometry reduce skin friction by stabilizing near-wall vortical motions [17,18]. Digital reconstructions of real denticles revealed effective control of vortex diffusion, confirming that biological micro-textures contribute to passive flow regulation [19]. Hydrodynamic performance varies with scale, since oversized denticles have been found to increase drag rather than reduce it, indicating a narrow optimal design window [20]. Two primary mechanisms have been proposed to describe the drag reduction effect: (i) the secondary vortex mechanism, where small-scale vortices suppress turbulence burst intensity [21,22], and (ii) the protruding height mechanism, where riblets increase viscous sublayer thickness and limit cross-flow momentum exchange [23,24]. Reported drag reduction values typically range between 5% and 12.8%, with a maximum of 35% achieved in specific experimental conditions using 3D printed denticles [20]. In addition to flow-control and drag-mitigation benefits, bio-inspired surface texturing has shown strong potential in anti-biofouling applications. Pulletikurthi et al. [25] demonstrated that shark-skin-inspired denticle structures can disrupt slime-layer adhesion by actively manipulating near-wall turbulence through unsteady blowing and suction effects. Their experimental visualization using PIV and microscopy confirmed that such surfaces inhibit the initiation and uniform spreading of biological films under both laminar and turbulent flow conditions. Since biofouling increases drag, energy consumption, and maintenance costs in marine systems, the capacity of biomimetic riblets to simultaneously manage hydrodynamic loads and suppress organism attachment highlights a promising multifunctional strategy for sustainable maritime performance.

Superhydrophobic structures inspired by lotus leaf, rice leaf, and water strider legs achieve drag reduction by trapping air within hierarchical microstructures. This forms a plastron layer that increases slip at the surface and decreases viscous resistance [26,27]. Experimental studies have reported drag reduction rates from 14% [28] to 50% in turbulent flows over structured coatings [29]. Results indicate that effectiveness depends strongly on microstructure dimensions and Reynolds number, since insufficient roughness scale reduces slip effect while over-extension may collapse the plastron [30,31]. The emerging combination of superhydrophobic coatings with riblet surfaces has further improved performance to 57%, highlighting synergy between flow separation delay and slip enhancement [32]. This technology has shown strong potential for marine applications, although durability and pressure resistance remain challenges.

Gill-inspired jetting provides an active mode of drag reduction by supplying momentum opposite to wall shear direction. Numerical and experimental studies have shown that jet flow can create a vortex pad effect, thickening the viscous sublayer and reducing local shear stress [33,34]. The injected flow also generates additional forward thrust that offsets frictional resistance [35]. Reported drag reduction rates are high when operating under optimized geometric and velocity conditions. For example, a reduction of 33% was achieved with controlled jet hole geometry [36], while 34% was obtained through multi-parameter optimization of jet diameter, angle, and velocity ratio [34]. These findings indicate promise for water-jet surfaces; however, their reliance on active energy input requires careful integration into propulsion and structural systems. Sharkskin-inspired surfaces demonstrate significant aerodynamic noise reduction in addition to drag mitigation. Experiments on cylinders with periodic denticle-like microstructures under turbulent inflow revealed a 2–6 dB (30–75%) decrease in overall noise compared to smooth baselines. These features attenuated large-scale vortex shedding and pressure fluctuations, especially in low-frequency ranges, without inducing flow separation [37]. This also points out the potential of bio-inspired texturing for passive aeroacoustic control in aircraft, UAVs, and industrial systems.

**Figure 2:**
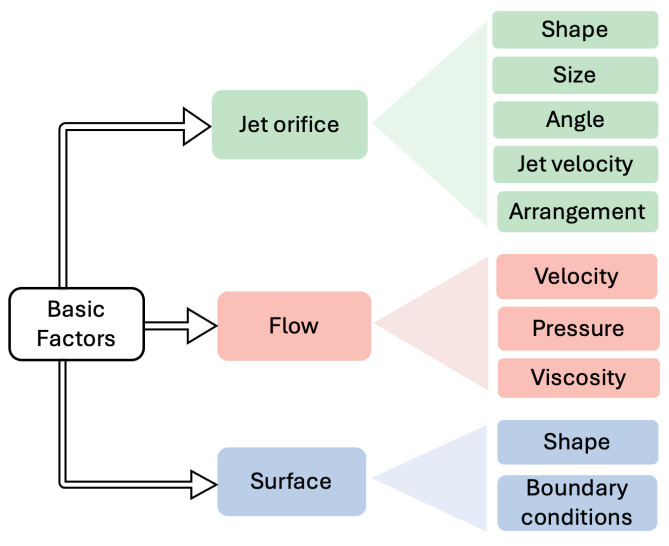
Key geometric, flow, and surface parameters governing biomimetic water-jet drag-reduction systems, adapted and redrawn from Liu et al. [38].

Active jet-based drag reduction strategies are highly sensitive to geometric, flow-governing, and surface-interaction parameters that dictate their overall performance. As shown in Figure 2, the characteristics of the jet, including its shape, size, inclination angle, jet velocity, and spatial arrangement, directly influence the momentum input into the boundary layer and the formation of coherent vortex structures. External flow-field properties such as free-stream velocity, local pressure distribution, and fluid viscosity determine how injected jets interact with the surrounding shear layers, thereby affecting the magnitude and stability of the vortex-pad effect. Furthermore, boundary-surface constraints, including surface curvature, substrate geometry, and embedded groove or pit configurations, alter the wall shear environment and modulate the entrainment capacity of the jet-induced vortical motion. The interplay of these parameters defines both the drag reduction potential and the required energy expenditure of biomimetic jet-based systems [38].

**Figure 3:**
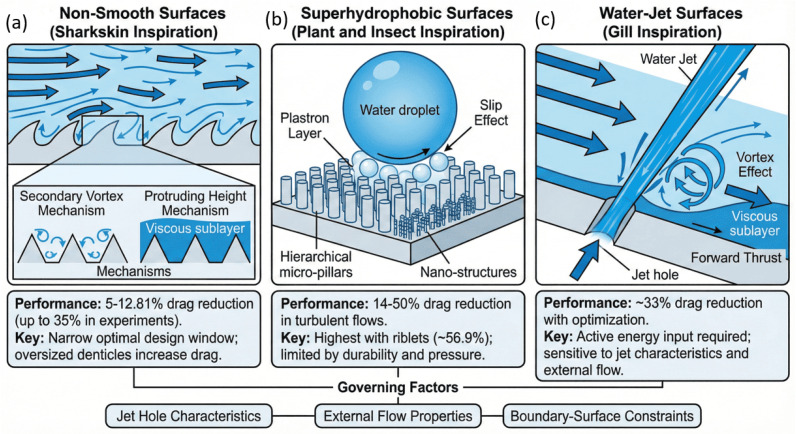
Bio-inspired drag-reduction technologies derived from marine and aquatic organisms: (**a**) sharkskin-inspired riblets that modulate near-wall vortices and viscous-sublayer geometry [17,18,20,22,23]; (**b**) superhydrophobic surfaces that use entrapped air layers to induce slip and reduce momentum transfer [26,29,31]; and (**c**) gill-inspired water-jet surfaces that inject momentum to thicken the viscous sublayer and lower shear stress [33,34]. Adapted from reported biological principles and drag-reduction trends.

Figure 3 and Table 1 summarizes the primary categories of bio-inspired drag-reduction surface strategies explored for underwater flow environments. Sharkskin-inspired riblet textures passively suppress turbulent momentum transfer by inducing secondary vortex stabilization and regulating viscous-sublayer thickness, thereby lowering skin friction without external energy input [17,18,20,22,23]. Superhydrophobic surfaces, drawing inspiration from micro/nano-structured leaf and insect cuticles, entrap a plastron layer that produces interfacial slip and reduces viscous drag when maintained under suitable pressure conditions [26,29,31]. Gill-inspired water-jet surfaces actively inject momentum into the boundary layer, creating vortex shielding and partial thrust that counteracts frictional resistance [33,34]. These mechanisms demonstrate how biological flow-control strategies can be translated into engineered textures and actuation concepts for substantial drag-reduction gains in marine applications.

Across riblet, superhydrophobic, and jet-based drag-reduction strategies, the governing physics can be unified using a small set of dimensionless parameters that control near-wall turbulence modification and energy exchange. For passive riblet surfaces, drag reduction appears maximized when the riblet spacing normalized by viscous length satisfies $s^{+}=su_{\tau }/\nu \approx 15-20$, with degradation occurring for $s^{+}>30$, where near-wall vortices penetrate the grooves and increase dissipation [39,40]. This scaling highlights that riblets primarily act by regulating coherent near-wall structures within the buffer layer, rather than altering outer-layer turbulence. Superhydrophobic surfaces introduce an additional slip-based mechanism governed by the ratio of slip length to viscous length, as well as capillary stability constraints characterized by Weber and capillary numbers [41]. Effective turbulent drag reduction requires that surface roughness remain below approximately half the viscous length scale and that capillary pressure exceed turbulent pressure fluctuations to preserve the air plastron. In contrast, active jet-based strategies operate through momentum injection and are governed by velocity ratio, jet Reynolds number, and momentum coefficient, trading higher drag reduction potential for increased energetic cost and system complexity. When viewed through this nondimensional framework, passive strategies primarily redistribute turbulent energy near the wall with minimal power input, whereas active strategies directly modify the momentum budget of the boundary layer. Biological inspiration therefore leads to new physics when it introduces qualitatively distinct transport pathways, such as slip-induced turbulence attenuation or vortex shielding, but constitutes incremental optimization when it primarily tunes geometry within established turbulent scaling limits [40].

Despite the advancements in sharkskin-inspired riblets, superhydrophobic coatings, and gill-based water-jet actuation, several challenges delay large-scale adoption of bio-inspired drag-reduction systems in real marine environments. The performance of riblet textures remains highly sensitive to geometric scaling and flow regime, and manufacturing biologically realistic microstructures over large hull areas with durability against abrasion and fouling is still unresolved. Superhydrophobic surfaces suffer from plastron collapse under hydrostatic pressure and long-term surface degradation, limiting their functionality in deep-water and high-speed applications. Active water-jet systems exhibit strong drag-reduction performance but require continuous energy input and mechanically complex integration, raising concerns about system reliability and net efficiency in practical vessels. Future efforts may therefore focus on hybrid passive–active designs that optimize riblet–air-layer synergy, development of anti-fouling and pressure-resistant surface chemistries, and closed-loop water-jet control strategies supported by low-power micro-pumps and onboard energy harvesting. Also, more comprehensive fluid-structure experiments and multi-scale simulations are needed to quantify performance under transient ocean conditions, enabling robust, economically viable deployment of adaptive drag-reduction systems in maritime transportation and underwater robotics.

**Table 1:** Comparison of bio-inspired marine drag-reduction strategies using governing mechanisms and nondimensional parameters.

Strategy	Primary Governing Mechanism	Key Dimensionless Parameters	Typical Drag Reduction and Main Limitation
Riblet surfaces	Near-wall coherent structure regulation and viscous sublayer thickening	Re, friction Reynolds number $Re_{\tau }$, riblet spacing in wall units $s^{+}$	5–15% typical, peak ∼35%; sensitive to $s^{+}$ and manufacturing scale [17,20,22,24,39]
Superhydrophobic surfaces	Slip-induced shear reduction with capillary-stabilized air plastron	$Re_{\tau }$, slip-length-to-viscous-length ratio, We, Ca	10–50% reported; plastron collapse under pressure and turbulent shear [28,29,39,41]
Water-jet surfaces	Momentum injection and vortex-pad formation in boundary layer	Jet Reynolds number $Re_{j}$, velocity ratio, momentum coefficient $C_{\mu }$	20–35%; requires continuous energy input and system integration [33,34,35,39]

### 3.2. Bio-Inspired Wave Energy Systems

**Figure 4:**
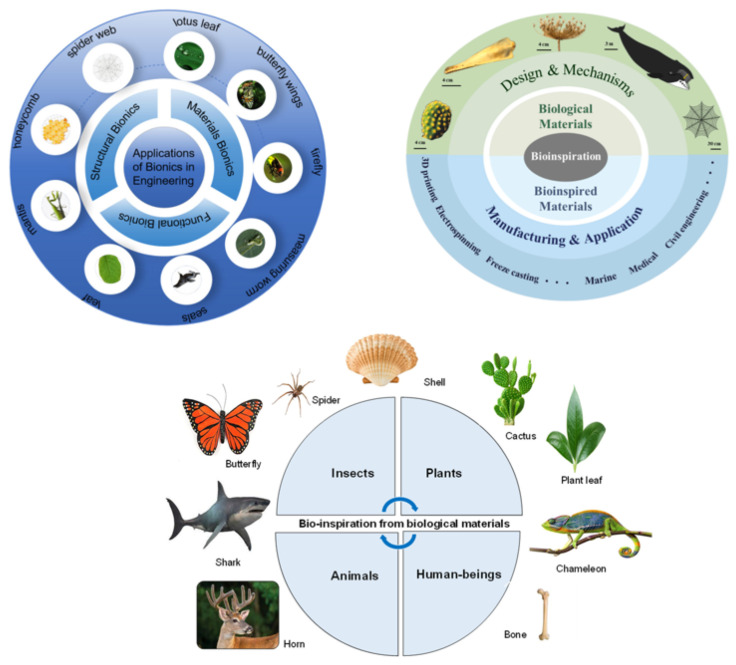
Practical engineering applications informed by biomimetic design concepts [42,43,44].

Bio-inspired wave energy converters have emerged as a promising class of technologies that leverage evolutionary hydrodynamic strategies to overcome the inherent limitations of conventional rigid-body WECs. Many marine organisms efficiently interact with unsteady ocean flows by stabilizing wake structures, suppressing drag-inducing separation, or harnessing vortex-induced oscillations for locomotion and thrust generation. Translating these mechanisms into engineering designs enables more effective coupling between wave kinematics and structural motion, particularly under low-velocity or highly variable sea states where traditional devices experience performance degradation. This section provides a systematic examination of the leading bio-inspired architectures reported in recent literature, with emphasis on the fluid mechanics principles that govern their enhanced energy capture efficiency, structural responsiveness, and operational adaptability in realistic ocean environments. Figure 4 provides a conceptual map of practical engineering applications of biomimetics.

**Figure 5:**
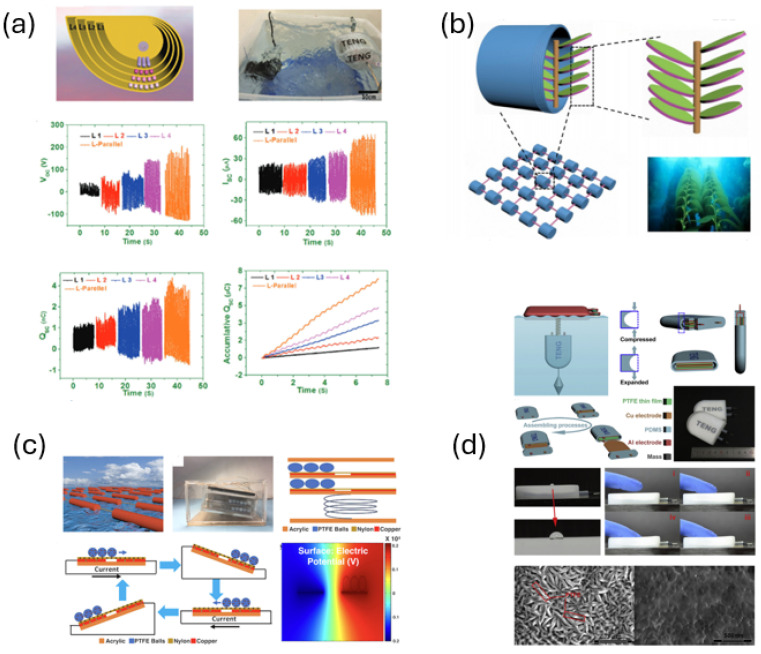
Bio-inspired wave energy harvesting concepts adapted from Zhang et al. [42]: (**a**) folding-wing converter inspired by flying-fish fins, where resonant flapping enhances lift–drag coupling; (**b**) dolphin-inspired streamlined geometry that suppresses adverse shedding and lowers hydrodynamic damping. These designs illustrate distinct hydrodynamic strategies that improve wave-induced force capture and energy conversion; (**c**) eel-like VIV device exploiting alternating vortex shedding for transverse oscillations in low-speed currents; and (**d**) scallop-shell flap with asymmetric curvature to increase pressure loading and reduce return-stroke losses.

Recent bio-inspired wave energy converters demonstrate that shaping and kinematic tuning of energy-harvesting structures can substantially enhance wave–structure coupling by manipulating unsteady hydrodynamic forces. Folding-wing and flapping-based designs inspired by flying-fish fins exploit resonant lift–drag oscillations to amplify energy transfer when the structural flapping frequency is matched to dominant wave periods, resulting in capture-width ratios approximately 30% higher than those of conventional point absorbers under comparable conditions [45]. Similarly, asymmetric flap geometries inspired by scallop shells increase pressure loading during wave impact while reducing return-stroke losses, thereby enhancing hydrodynamic moments and improving capture factors by about 30% relative to rectangular flaps [46]. In contrast to resonance-based strategies, elongated eel-inspired devices deliberately harness vortex-induced vibrations to sustain transverse oscillations in weak currents, enabling effective power extraction at flow velocities around 0.4 m/s, well below the operational thresholds of traditional electromagnetic generators [47]. Streamlined dolphin-inspired hulls follow a complementary approach by suppressing detrimental vortex shedding and reducing hydrodynamic damping, which increases the fraction of incident wave energy converted into mechanical motion and yields measurable gains in both hydraulic and electromechanical efficiency [48]. These systems illustrate two dominant bio-inspired pathways for wave energy enhancement: either suppressing unfavorable flow separation to reduce energy losses or deliberately exploiting unsteady vortex dynamics to amplify structural response under low-velocity or highly variable sea states. Figure 5 evaluates bio-inspired strategies that improve wave energy capture by either suppressing adverse vortex shedding or exploiting vortex-induced oscillations.

Bio-inspired triboelectric, piezoelectric, and hybrid wave energy harvesters consistently demonstrate that structural compliance, geometric adaptability, and nonlinear dynamics play a central role in enhancing fluid–structure interaction under low-velocity and broadband excitation conditions. Devices inspired by jellyfish bells, sea snakes, kelp blades, and soft fins employ compliant or segmented morphologies that conform to incident wave kinematics, reduce vortex-induced resistance, and stabilize hydrodynamic loading, thereby enabling sustained oscillatory motion where rigid structures perform poorly [49,50,51,52]. Complementary strategies implements nonlinear dynamics, such as bistable mechanisms and vortex-induced vibrations, to trigger large-amplitude responses over extended frequency ranges, substantially outperforming linear harvesters in unsteady flows [53]. Hybrid configurations that combine triboelectric and electromagnetic conversion further improve robustness and operational bandwidth by converting fluctuating lift and drag forces into compounded mechanical responses, enabling effective energy harvesting at current speeds below 0.3 m/s [54,55,56]. These studies indicate that bio-inspired energy harvesters derive their advantage not from absolute electrical output under laboratory conditions, but from enhanced hydrodynamic adaptability, reduced sensitivity to flow variability, and improved coupling between unsteady fluid forces and structural motion. Table 2 summarizes the dominant bio-inspired design strategies applied in hydrodynamic energy harvesting devices and their associated performance improvements.

**Figure 6:**
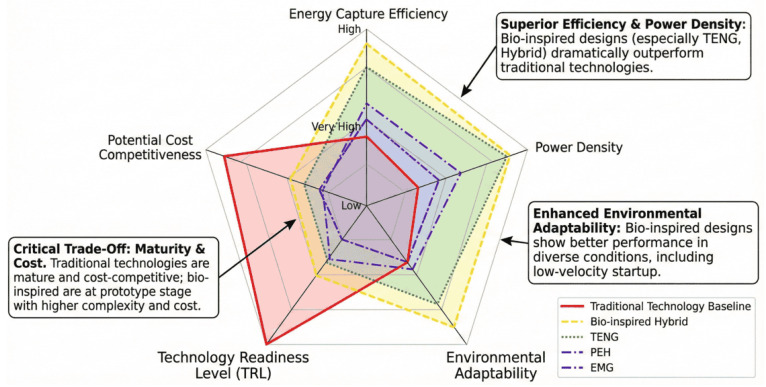
Comparison of bio-inspired and traditional WECs in efficiency, power density, environmental adaptability, readiness level, and cost potential [42].

Figure 6 compares bio-inspired wave energy converters with conventional devices using normalized metrics of energy capture efficiency, power density, environmental adaptability, technology readiness level, and cost potential. Hybrid bio-inspired systems exhibit a balanced performance profile, combining relatively high power density and adaptability with competitive efficiency. Triboelectric-based devices show strong adaptability to low-velocity conditions but remain limited by low technology readiness, while piezoelectric-based systems display moderate performance across metrics. Bio-inspired electromagnetic converters approach commercial readiness but generally exhibit lower capture efficiency under weak or variable sea states. In contrast, conventional WECs retain higher maturity and cost competitiveness but perform poorly in low-velocity and highly unsteady environments, indicating that fluid-mechanics-driven biomimicry primarily improves adaptability and energy capture where rigid-body designs are constrained.

A unifying feature of bio-inspired fluid systems is the dependence of wake topology and force generation on non-dimensional scaling laws. Research indicates that efficient momentum transfer whether for propulsion or energy extraction requires synchrony between frequency, amplitude, and flow speed, typically manifesting as a Strouhal number in the range of St≈0.2−0.3 [58]. This scaling provides a metric for evaluating bio-inspired WECs. When WEC kinematics align with these biologically preferred regimes, the system exploits coherent reverse von Kármán vortex streets to enhance energy capture efficiency. Conversely, deviations lead to disparate wake patterns and increased drag [59]. Thus, the Strouhal number and reduced frequency act as primary predictors of WEC performance, offering a physics-based lens to rationalize the effectiveness of bio-inspired designs across varying morphologies.

Despite promising gains in capture width ratio, power density, and low-velocity start-up, bio-inspired wave energy converters remain constrained by several practical challenges. Most reported devices are validated under idealized laboratory waves or 2D simulations, so their performance and survivability in irregular, multi-directional seas, with strong Reynolds-number and scale effects, are still uncertain [42]. Long-term durability under corrosion, biofouling, and material fatigue is also poorly quantified, and there is limited assessment of mechanical, hydraulic, and electrical losses or of levelized cost of energy, which restricts fair comparison with mature conventional WECs. Future work may prioritize coupled fluid–structure–electrical modeling and uncertainty analysis, extended sea trials for representative bio-inspired EMG, TENG, PEH, and hybrid systems, and systematic techno-economic studies that include operations and maintenance. In parallel, progress in anti-fouling and high-strength soft materials, robust sealing and encapsulation for TENGs, and modular array layouts optimized for environmental loading and power smoothing will be essential to translate the rich biological design space into scalable, bankable wave energy technologies.

### 3.3. Bio-Inspired Aerospace Design

**Figure 7:**
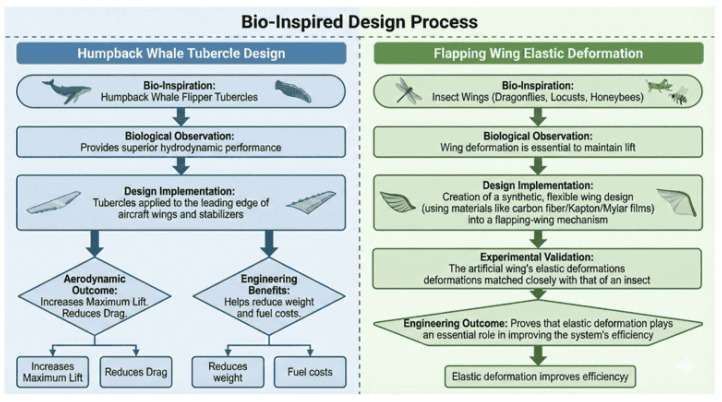
Bio-inspired aerodynamic design strategies that translate natural flow-control mechanisms into enhanced lift, reduced drag, and improved unsteady-flight efficiency in engineered wings [60,62,63].

The application of bio-inspired wing architectures demonstrates that aerodynamic performance improvements across multiple flight regimes primarily arise from passive manipulation of unsteady vortex dynamics and controlled fluid–structure interaction. Morphing and flapping wings inspired by avian and insect flight enable adaptive planform geometry and dynamic camber variation, which delay flow separation and sustain leading-edge vortices under low-Reynolds-number and unsteady conditions [60,61,62]. Similarly, leading-edge tubercles derived from humpback whale flippers act as passive vortex generators that energize the boundary layer and extend the post-stall operating envelope, although their benefits remain regime dependent and may introduce drag penalties outside high-angle-of-attack conditions [63]. Figure 7 illustrates how bio-inspired aerodynamic concepts, including humpback-whale tubercles and insect-wing elastic deformation, translate natural flow-control mechanisms into enhanced lift, reduced drag, and improved unsteady-flight efficiency.

**Figure 8:**
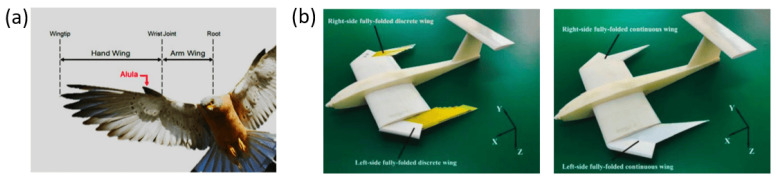
Bio-inspired aerodynamic wing designs guided by the alula mechanism where (**a**) deployed alula on a bird wing [66] and (**b**) wind tunnel test models featuring discrete and continuous bio-inspired wing designs [66]. The alula, located near the wrist joint of bird wings, delays stall and maintains flow attachment during high-angle maneuvers. Inspired by this biological control feature, both discrete and continuous alula-based wing designs have been engineered and experimentally evaluated to quantify improvements in post-stall stability and aerodynamic performance Budholiya et al. [66].

Beyond continuous leading-edge modifications, discrete flow-control devices inspired by natural feather structures further improve post-stall performance. Alula-inspired leading-edge devices suppress separation and promote lift recovery in post-stall and deep-stall regimes by locally stabilizing the shear layer near the wing root [64,65]. Experimental comparisons between discrete and continuous alula-based designs indicate that sectionalized surfaces can improve lateral stability and gust tolerance, although the associated aerodynamic gains remain sensitive to Reynolds number and deployment configuration [66]. Figure 8 illustrates bio-inspired wing designs guided by the alula mechanism, showing how discrete and continuous leading-edge devices delay stall and maintain flow attachment during high-angle maneuvers.

**Figure 9:**
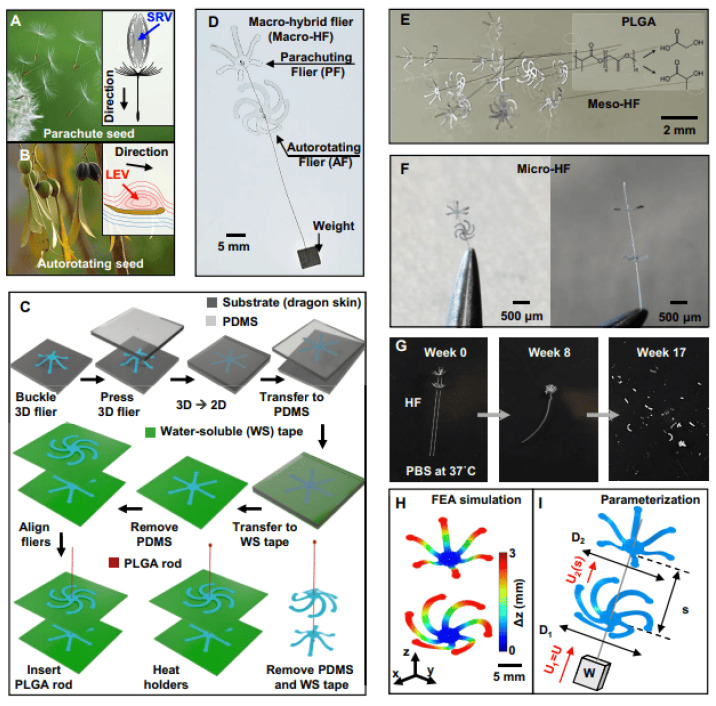
Hybrid bio-inspired fliers combining multiple biological vortex mechanisms. (**A**,**B**) Natural seed archetypes demonstrating separated vortex-ring (SVR) formation in dandelions and leading-edge vortex (LEV) stabilization in autorotating samaras. (**C**) Buckling-guided fabrication workflow enabling tunable 3D geometries. (**D**–**F**) Geometric scalability from macro- to microscale architectures with integrated ballast or PLGA rods. (**G**) Time-dependent bioresorption in PBS at 37 °C demonstrating environmental compatibility. (**H**,**I**) Finite-element simulation and geometric parameterization outlining the aerodynamic design space. Adapted with permission from Kim et al. [67].

Recent progress in bio-inspired aerodynamics moves beyond single-mechanism optimization toward hybrid architectures that integrate multiple passive vortex strategies within a unified structure. Hybrid mesoscale fliers inspired by dandelion seeds and auto-rotating maple samaras simultaneously generate separated vortex rings and stabilized leading-edge vortices, enabling enhanced aerodynamic loading, prolonged descent times, and improved passive stability relative to single-strategy designs [67]. Coupled particle image velocimetry, numerical and analytical modeling demonstrate constructive vortex interaction and provide drag-scaling relationships applicable to micro-aerial vehicle design. These hybrid concepts introduce qualitatively new aerodynamic pathways rather than incremental geometric tuning, although their performance remains sensitive to Reynolds-number scaling, fabrication tolerances, and structural robustness. Figure 9 shows hybrid bio-inspired fliers that combine multiple biological vortex mechanisms, demonstrating scalable architectures that leverage separated vortex-ring formation and leading-edge vortex stabilization for enhanced aerodynamic performance.

Despite demonstrated aerodynamic improvements, bio-inspired wing architectures face persistent challenges that must be addressed before widespread aerospace deployment. Manufacturing complex morphing or corrugated geometries at aircraft-relevant scales remains difficult, particularly when simultaneously targeting low structural mass, high fatigue resistance, and aero-elastic stability. Performance trade-offs also emerge leading-edge tubercles may reduce stall but can increase drag in cruise regimes, and flexible wings improve maneuverability yet introduce control uncertainty and structural reliability concerns under gust loading. From a systems perspective, integrating distributed sensing, actuation, and control without penalizing weight or energy consumption remains a major bottleneck. Future efforts may focus on multi-functional structural materials, fluid–structure interaction optimization, and digital design frameworks that combine bio-inspired flow physics with real-time adaptive morphing. Advances in additive manufacturing, 4D-printed smart materials, and data-driven aerodynamic control are expected to accelerate translation into practical micro-air-vehicles and next-generation sustainable aircraft systems, where biologically informed designs can provide improved efficiency, maneuverability, and operational resilience.

### 3.4. Corrugated Wing Aerodynamics

Corrugated airfoils inspired by insect wings, particularly those of dragonflies, exhibit robust aerodynamic performance in low-Reynolds-number regimes where conventional smooth airfoils suffer early separation and stall. The primary fluid-mechanical mechanism arises from the formation of quasi-steady recirculation bubbles within the corrugation troughs, which act as a virtual aerodynamic contour that stabilizes the shear layer and sustains lift at moderate angles of attack [68,69,70]. These trapped vortical structures reduce sensitivity to surface roughness and geometric imperfections, contributing to stable force generation under unsteady inflow conditions.

Additional performance gains emerge when corrugated wings exhibit passive aeroelastic deformation, allowing dynamic adaptation of camber and load distribution in response to fluctuating aerodynamic forces [71,72]. Tandem corrugated configurations further enhance lift generation through constructive wing–wake interactions, enabling sustained hovering and improved propulsive efficiency in bio-inspired micro air vehicles [73,74]. These benefits highlight that corrugation primarily functions as a vortex-management strategy rather than a means of reducing viscous drag.

Despite these advantages, corrugated wings present notable limitations that constrain their broader application. Corrugations often increase drag at low angles of attack, making their aerodynamic benefit strongly dependent on operating regime and geometric tuning [75]. Structural scalability also remains challenging, as replicating the stiffness-to-weight characteristics of insect venation at larger scales requires advanced materials and fabrication techniques. Consequently, current research emphasizes high-fidelity fluid–structure interaction modeling and additive manufacturing approaches to tailor corrugation wavelength, amplitude, and flexibility for specific mission profiles, while mitigating drag penalties outside the low-Reynolds-number envelope [76].

**Figure 10:**
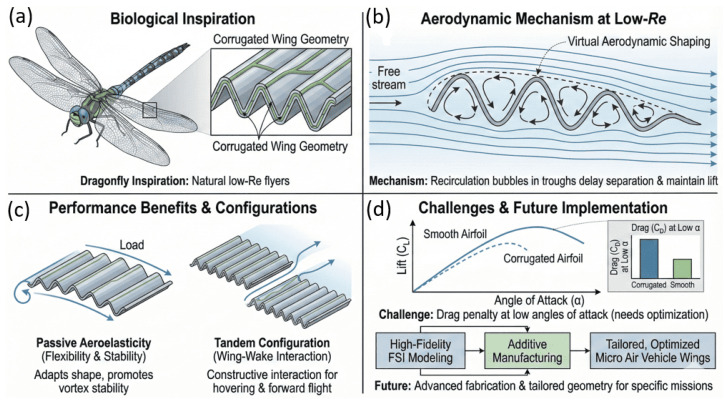
Overview of bio-inspired corrugated-wing aerodynamics and associated design pathway. (**a**) Biological inspiration from dragonfly wings highlighting corrugated geometries effective in low-Reynolds-number flight. (**b**) Low-Re aerodynamic mechanism where recirculation bubbles within corrugation troughs delay separation and maintain lift. (**c**) Performance benefits of corrugated wings, including passive aeroelastic adaptation and tandem wing–wake interaction. (**d**) Trade-offs and implementation challenges, showing lift enhancement with drag penalties at low angles of attack and the need for optimized fabrication [73,75,76].

Figure 10 summarizes the biological inspiration, governing flow mechanisms, and key performance trade-offs associated with corrugated wings, illustrating how vortex stabilization, aeroelastic adaptation, and wing–wake interaction enable effective low-Reynolds-number flight while highlighting the need for regime-aware design optimization.

Despite strong evidence that corrugated wings enhance lift, delay stall, and stabilize flow in low-Reynolds-number regimes, several important challenges remain for practical deployment. Corrugations can increase drag at low angles of attack, requiring optimization of wavelength, amplitude, and location to ensure performance robustness across variable operating conditions. Structural scalability is another limitation: dragonfly-like vein-reinforced morphologies are difficult to replicate using conventional manufacturing, and achieving their high stiffness-to-weight ratios demands advanced materials and fabrication strategies. Also, most aerodynamic benefits diminish at higher Reynolds numbers, highlighting the need to clarify operating envelopes for micro air vehicles and marine systems. Future efforts can consider high-fidelity fluid–structure interaction modeling, hybrid smooth-corrugated designs, and adaptive flexibility to actively tune wake interactions. Emerging directions noted in the review include integrating smart materials, additive manufacturing, and onboard sensing to create morphing wings that autonomously adjust geometry for maximal aerodynamic efficiency across flight regimes.

### 3.5. DRL-Enabled Bio-Inspired Fluid Control

Recent progress in bio-inspired fluid mechanics has increasingly incorporated deep reinforcement learning (DRL) to capture the adaptive behaviors observed in biological swimmers and flow-interacting structures. DRL provides a powerful alternative to traditional control or shape-optimization methods due to its ability to interact with nonlinear, high-dimensional fluid environments and learn optimal policies through repeated environment interaction.

**Figure 11:**
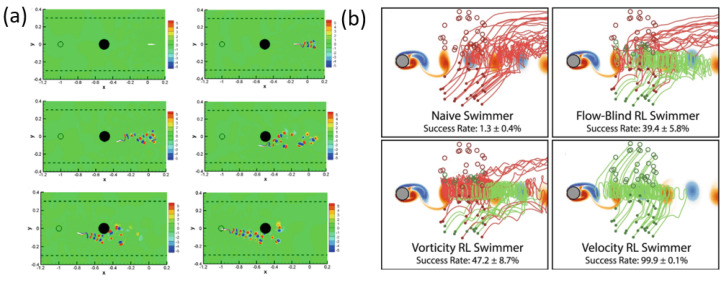
Deep reinforcement learning strategies for bio–inspired swimmers operating in complex fluid environments. (**a**) Representative swimmer trajectories and flow-field evolution during reinforcement–learning–based navigation in a complex vortical environment. (**b**) Comparison of swimmer control strategies, showing improved navigation success from naive to flow–aware reinforcement–learning swimmers [81,82].

DRL can uncover energy-efficient swimming strategies analogous to those evolved in fish schools. Gazzola et al. [77] demonstrated that a single swimmer controlled by Q-learning could self-propel effectively in a viscous flow, while Novati et al. [78] showed that a follower fish could increase swimming efficiency by approximately 20% by exploiting the vortex wake of a leader. This coordinated swimming behavior, further validated in three-dimensional formations using LSTM-enhanced DQN networks [79], illustrates how DRL enables collective swimmers to harness environmental vortices for reduced energy expenditure as shown in Figure 11. DRL has also enabled intelligent navigation in microscale unsteady environments, where the flow is chaotic and difficult to predict. Studies by Amoudruz and Koumoutsakos [80] and Gustavsson et al. [81] showed that microswimmers trained with actor–critic or Q-learning methods can outperform naïve swimmers when navigating toward targets or escaping fluid traps, demonstrating decision-making capabilities adapted to turbulent eddy structures as illustrated in Figure 11.

In addition, DRL contributes to the control of bio-robotic locomotion. Mirzakhanloo et al. [83] trained agents to minimize hydrodynamic disturbance fields, thereby achieving active fluid-mediated invisibility for predator-avoidance and stealth pursuit. Reinforcement-learning has also been used to discover robust gliding behaviors in deformable ellipsoidal bodies for either minimum-energy or minimum-time descent, outperforming model-based optimal control and aligning with biological strategies such as foraging and escape [78]. Shape adaptation is another critical direction in bio-inspired mechanics. Viquerat et al. [84] used DRL to optimize Bessel-curve-based aerodynamic shapes that maximize lift-to-drag ratio, autonomously producing airfoil-like morphologies under Reynolds numbers of a few hundred.

**Figure 12:**
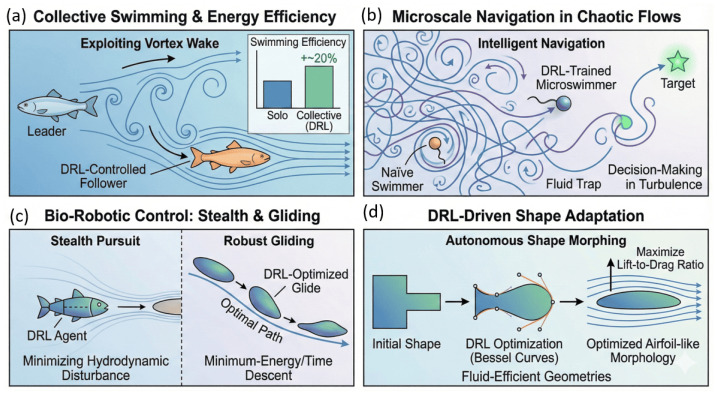
Summary of deep reinforcement learning (DRL) applications in bio-inspired fluid mechanics. (**a**) DRL-enabled collective swimming exploiting wake interactions to improve energy efficiency. (**b**) DRL-based microswimmer navigation enabling target-seeking in chaotic flow fields. (**c**) Bio-robotic control using DRL for stealth pursuit and energy-efficient gliding. (**d**) DRL-driven autonomous shape adaptation toward fluid-efficient airfoil-like geometries [77,78,79,80,81,83,84].

The DRL has then enabled the development of bio-inspired swimmers and deformable structures that intelligently interact with fluid environments. As illustrated in Figure 12, DRL agents can autonomously discover efficient propulsion strategies, such as energy-saving wake exploitation in leader–follower swimming formations [77,78,79]. At the microscale, DRL-trained swimmers outperform naïve swimmers by successfully avoiding fluid traps and navigating toward dynamic targets in chaotic flow fields [80,81]. DRL frameworks have also been applied to bio-robotic control where agents minimize hydrodynamic disturbances for stealth pursuit and optimize glide trajectories to reduce locomotion cost [78,83]. In addition, DRL-based shape optimization enables continuous body morphing that leads to fluid-efficient geometries with improved lift-to-drag performance [84].

Although DRL has demonstrated strong potential in bio-inspired fluid mechanics, several limitations currently restrict broader adoption. Most demonstrations remain simulation-only, and performance rarely transfers reliably to real-world robotic systems due to model uncertainties and sensing noise. Training remains computationally expensive, as thousands of coupled CFD–learning iterations are required, underscoring the need for surrogate-based or reduced-order approaches. In addition, existing methods depend on partial or simplified flow observations, limiting the agent’s ability to exploit full fluid-structure coupling. Standardized benchmarks and reproducibility practices are also lacking, making cross-study comparison difficult. Addressing these challenges will require improved physical sensing integration, more efficient training algorithms, and validated frameworks that operate robustly in experimental environments [12].

## 4. On Bio-Inspired Fluid–Structure Interaction

Fluid–Structure Interaction (FSI) is a central mechanism behind efficient biological motion. Organisms such as fish, insects, jellyfish, and flexible plants use controlled compliance to manage vortex formation, reduce drag, and enhance thrust, which enables high performance in unsteady environments. Bio-inspired engineering simplifies these natural mechanisms into flexible foils, membranes, and filaments to reveal how material stiffness, geometry, and motion influence wake structure and flow energy transfer [85]. Adapting structure in response to flow conditions can improve propulsion, mixing, and even thermal transport. However, current bio-inspired heat-transfer surfaces generally rely on rigid geometries, which limits their ability to sustain surface renewal or respond to dynamic bubble and droplet behavior. Integrating FSI concepts into boiling and condensation surfaces therefore presents a clear opportunity for improved droplet or bubble removal, reduced fouling, and more stable high-performance heat transfer [86].

Recent investigations into bio-inspired FSI further highlight how biological systems couple flexibility, kinematics, and vortex dynamics to deliver multifunctional performance enhancements across propulsion, flow control, and energy exchange. Wang et al. [86] demonstrates that simplified flexible slender structures derived from locomotion strategies of fish and insects (flapping-foil propulsion), jellyfish and cephalopods (jetting/paddling), and micro-organisms (ciliary beating) which enable high thrust efficiency by regulating wake topology and vortex phasing, while a careful balance of flexibility can either suppress or enhance reverse Kármán street symmetry depending on operational objectives [87,88,89,90].

Similarly, plant-inspired wall-mounted filaments achieve multiple fluidic functions including mixing enhancement, drag suppression, and convective heat-transfer augmentation through passive reconfiguration and VIV-driven interaction modes [91,92,93,94]. Flexible plates with tailored mass ratio and piezoelectric coupling have also shown turbine-comparable energy-harvesting capabilities under flutter-dominated conditions [95,96,97,98]. These studies emphasize that biological architectures inherently optimize structural compliance and hydrodynamic feedback properties that remain underexplored in current bio-inspired thermal management systems that predominantly rely on static geometric texturing. This reveals a key research gap: the lack of adaptive, FSI-enabled surface-flow coupling in phase-change heat-transfer designs, where incorporating compliant microstructures, distributed sensing, and data-driven control strategies could unlock dynamic bubble/droplet regulation, enhanced heat-transfer headroom, and improved resilience under transient energy loads.

**Figure 13:**
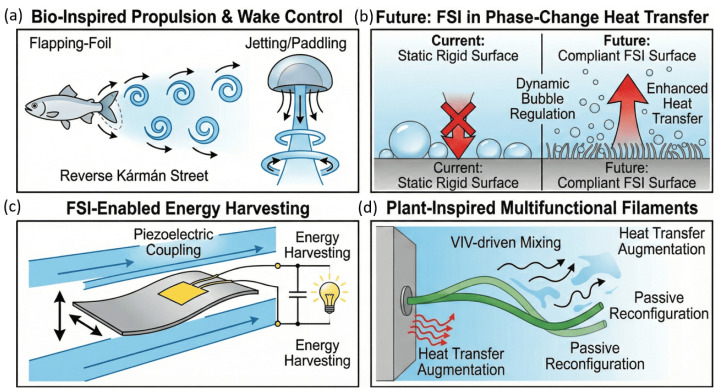
Conceptual overview of bio-inspired fluid–structure interaction systems and their translation into thermal–fluid applications. (**a**) Bio-inspired propulsion mechanisms using flapping and jetting to regulate wake structure and momentum transfer. (**b**) Conceptual transition from rigid to compliant FSI surfaces enabling dynamic bubble regulation and enhanced phase-change heat transfer. (**c**) FSI-enabled energy harvesting through fluid-driven deformation and piezoelectric coupling. (**d**) Plant-inspired flexible filaments providing passive reconfiguration for mixing enhancement and heat-transfer augmentation [86].

Figure 13 summarizes how biological Fluid–Structure Interaction (FSI) mechanisms translate into multifunctional engineering applications. Flapping-foil propulsion in aquatic and aerial organisms regulates vortex formation and wake topology to enhance thrust efficiency [87,88,89,90]. Jetting and paddling motions used by jellyfish and cephalopods illustrate how compliant body deformation can produce efficient momentum exchange with the surrounding fluid [86]. FSI-enabled structural compliance has also been utilized in piezoelectric energy harvesting, where fluid-driven fluttering of flexible plates converts kinetic energy into electrical output [96,97]. Plant-inspired filaments undergo passive reconfiguration and VIV-driven motion, which promotes enhanced mixing and heat-transfer augmentation [92,93]. These examples highlight that biological flexibility and flow interaction produce adaptive and efficient performance in unsteady conditions. The figure also emphasizes that applying similar compliance-driven FSI concepts in boiling and condensation systems offers an opportunity to overcome the limitations of static rigid heat-transfer surfaces and enable dynamic bubble or droplet regulation for higher thermal performance [86].

Despite rapid progress, key challenges remain in advancing bio-inspired fluid–structure interaction and data-driven control. Various studies still rely on 2D numerical models, whereas biological performance is inherently 3D and multiscale, making simulations computationally intensive and technically difficult to parallelize efficiently. Moreover, design principles such as structural resonance and wake-resonance coupling in flapping propulsion are not yet fully understood, especially regarding their role in thrust efficiency and vortex stability. The onset of symmetry-breaking instabilities in flexible wakes also lacks a clear mechanistic explanation, requiring advanced global-stability analysis for resolution. At the microscale, realistic 3D ciliary coordination remains challenging due to biological complexity and non-Newtonian fluid behavior, which is still poorly captured in current models. From a control perspective, data-driven bio-inspired flow manipulation is limited by sparse sensing and low-dimensional observations. DRL applications still struggle with extracting richer flow-field features and transferring learned strategies from simulations to experiments. In addition, large-scale computation demands continue to constrain training throughput and algorithmic exploration, highlighting the need for more efficient CFD, parallelization, and robust neural architectures.

## 5. On Rheological Effects in Bio-Inspired Fluid–Structure Interaction

Many biological fluids relevant to bio-inspired systems exhibit non-Newtonian behavior, including shear-thinning viscosity, yield stress, and finite elastic relaxation times. Representative examples include mucus layers, biomass slurries, polymeric gels, and bioinks. These rheological properties fundamentally alter momentum transport, stress distribution, and mixing efficiency compared with Newtonian assumptions. Numerical investigations of intestine-inspired flexible peristaltic bioreactors demonstrate that shear-dependent viscosity alone can induce periodic local viscosity reductions exceeding 20%, leading to substantial enhancements in mixing, substrate conversion, and reaction rates relative to Newtonian-flow reactors [99]. In bio-inspired systems involving flexible filaments, flapping wings, or compliant walls, viscoelastic stresses introduce an additional time scale associated with fluid relaxation. When the actuation time scale approaches the relaxation time, elastic normal stresses modify wake structure, force generation, and stability characteristics. As a result, Newtonian-based analyses may misrepresent both performance gains and stability limits in biologically relevant environments dominated by viscoelastic fluids.

Beyond bulk rheology, biological interfaces often exhibit interfacial viscoelasticity due to polymeric or mucus-like layers adsorbed at solid–fluid boundaries. Bio-inspired liquid-infused surfaces highlight the importance of this effect. Studies of mucus-infused, villi-inspired surfaces show that drag is governed not only by surface texture and wettability, but also by elastic stress accumulation within the infused layer [100]. In these systems, drag reduction or enhancement is strongly dependent on deformation rate and elastic relaxation, rather than surface chemistry alone. These results extend classical wettability-based interpretations of slippery surfaces by demonstrating that interfacial mobility is controlled by stress relaxation dynamics. Interfacial viscoelasticity therefore represents a key, yet often overlooked, design parameter for bio-inspired drag control, antifouling, and interfacial transport applications.

Rheological effects are also relevant to bio-inspired thermal transport systems, particularly under boiling and condensation conditions. In non-Newtonian or polymeric liquids, temperature-dependent viscosity and relaxation times can vary sharply within microlayers adjacent to heated surfaces, influencing nucleation, microlayer stability, and bubble or droplet detachment dynamics. Despite this, most bio-inspired phase-change studies assume Newtonian working fluids. Evidence from bio-inspired reactor studies indicates that viscosity evolution during reaction or heating feeds back into transport performance by modifying local shear, mixing, and mass transfer [99]. Extending these concepts to thermal systems suggests that rheology-aware models are necessary for accurately predicting boiling inception, critical heat flux, and condensation efficiency when non-Newtonian fluids are involved.

To systematically incorporate rheological effects into bio-inspired performance evaluation, additional dimensionless groups must complement classical Reynolds, Weber, and Capillary numbers. The Weissenberg number $\mathrm{We}=\frac{\lambda U_{C}}{D}$, and the Deborah number $\mathrm{De}=\frac{\lambda \eta }{\rho L^{2}}$, quantify the relative importance of elastic stress relaxation compared with deformation rate or observation time, respectively. These parameters govern drag modulation, stress localization, and flow reorganization in viscoelastic flows over bio-inspired textured and liquid-infused surfaces [100].

When coupled with structural compliance, viscoelastic fluids introduce a tightly coupled fluid–structure–rheology interaction. Surveys of locomotion and transport in yielding and non-Newtonian substrates demonstrate that many biological systems exploit this coupling to achieve effective propulsion and maneuverability in environments inaccessible to rigid or Newtonian-based designs [101]. Incorporating rheology-specific dimensionless groups into bio-inspired design frameworks therefore provides a unified basis for comparing Newtonian and non-Newtonian operating regimes across flow control, fluid–structure interaction, and thermal transport applications.

## 6. On Bio-Inspired Droplet Condensation

Condensation heat transfer occurs primarily in two modes: filmwise and dropwise. In filmwise condensation, the condensate completely wets the surface and forms a continuous liquid film, introducing a substantial thermal resistance and yielding relatively low heat transfer coefficients [102,103,104]. In contrast, dropwise condensation proceeds through discrete droplets that grow, coalesce, and depart, repeatedly exposing fresh surface area. By minimizing the conduction path through the condensate and maintaining intermittent vapor–solid contact, dropwise condensation can achieve heat transfer coefficients an order of magnitude higher than filmwise condensation [105,106,107].

The condensation mode is governed by surface wettability and interfacial energy balance. On smooth surfaces, low equilibrium contact angles favor liquid spreading and film formation, while increasing hydrophobicity promotes droplet formation and dropwise behavior [108,109]. Real surfaces are textured, and their wetting response is commonly described by the Wenzel and Cassie–Baxter states. In the Wenzel state, liquid fully penetrates surface roughness, increasing adhesion and favoring filmwise wetting. In contrast, the Cassie–Baxter state involves trapped air pockets that reduce solid–liquid contact, lower adhesion, and enable droplet mobility [110,111]. Sustained dropwise condensation requires maintaining this metastable Cassie–Baxter state, while transition to Wenzel wetting leads to probable filmwise behavior.

The condensation heat flux depends on the characteristic droplet size and removal rate. Smaller droplets provide shorter conduction paths through the liquid, so surfaces that promote rapid droplet departure maintain higher average heat transfer coefficients [112]. Although hydrophilic surfaces reduce the nucleation energy barrier, they favor film formation; hydrophobic and textured surfaces increase the barrier but support isolated nucleation sites that evolve into discrete droplets [107]. Hierarchical micro- and nanostructures reconcile this tradeoff by enabling nucleation while preserving low adhesion and droplet mobility [113].

As droplets coalesce, the reduction in liquid–vapor interfacial area releases surface energy that can drive spontaneous droplet jumping or sliding when it exceeds adhesion and viscous losses. This self-propelled droplet removal prevents surface flooding and sustains high condensation heat transfer performance [105,106,114]. Overall, filmwise and dropwise condensation represent competing interfacial energy configurations, and hierarchical textured surfaces stabilize the dropwise mode by promoting low adhesion, rapid droplet removal, and sustained heat transfer enhancement [108].

**Figure 14:**
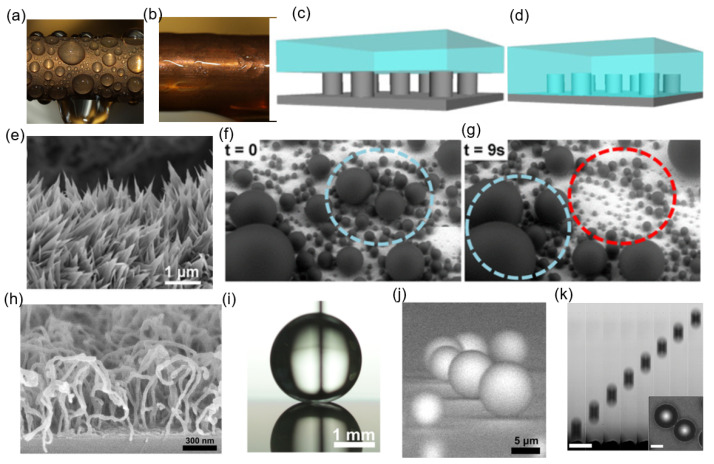
Overview of condensation modes, wetting states, and engineered micro/nanostructures that enable sustained dropwise condensation [105,115]. (**a**) Dropwise condensation on a hydrophobic surface and (**b**) filmwise condensation on a hydrophilic surface. (**c**) Schematic of the Cassie–Baxter wetting state, where droplets rest on top of surface asperities, and (**d**) the Wenzel wetting state, in which liquid penetrates surface texture. (**e**) SEM image of a CuO nanostructured surface grown for 10 min. (**f,g**) Time-lapse ESEM images showing droplet nucleation, growth, and coalescence-induced jumping during steady-state water condensation; a large viewing area was used to minimize electron-beam heating, and repeated surface renewal events are observed. (**h**) SEM image of a hydrophobic CNT “turf” coated with a P2i layer. (**i**) Macroscopic receding droplet on the CNT surface with an apparent contact angle of 166°±2°. (**j**) ESEM image of condensed droplets captured at an 8° tilt angle showing early-stage coalescence dynamics. (**k**) High-speed time-lapse sequence of coalescence-induced jumping ($T_{\mathrm{WW}}=5\;{}^{\circ}$C, $p_{v}=0.87$ kPa, shutter speed 185μs), with inset showing pre-jump droplets of radii $R_{1}=20.2\;\mu$m and $R_{2}=19\;\mu$m forming a merged droplet of radius $R_{j}=24.7\;\mu$m.

Figure 14 provides an overview of condensation modes, wetting states, and engineered micro or nanostructures that enable sustained dropwise condensation. Figure 14a,b depicts the contrasting heat-transfer behaviors of dropwise and filmwise condensation, where discrete droplets on a hydrophobic metallic surface rapidly nucleate, grow, and depart-continuously renewing the cold surface-while a continuous condensate layer on a hydrophilic surface introduces additional thermal resistance and suppresses heat transfer. Figure 14c,d shows schematics of the Cassie-Baxter and Wenzel wetting states, respectively, highlighting how air entrapment within surface asperities reduces solid-liquid contact area to stabilize mobile droplets in the Cassie-Baxter regime, in contrast to liquid penetration that increases adhesion and promotes pinned or filmwise behavior in the Wenzel state. Figure 14e–g demonstrates the role of nanoscale surface structuring in enabling efficient droplet dynamics: SEM imaging of CuO nanoflakes (e) and environmental SEM time-lapse frames (f,g) reveal high nucleation site density, coalescence-driven removal of droplets, and passive surface renewal through spontaneous jumping events. The hydrophobic carbon-nanotube (CNT) turf shown in Figure 14h, combined with the macroscopic receding angle of ∼166° in Figure 14i, indicates ultralow adhesion favorable for rapid droplet departure. Finally, Figure 14j,k shows coalescence-induced droplet motion, where asymmetric collapse and surface energy release propel droplets away from the surface; high-speed imaging confirms energy conversion during merging of microscale droplets ($R_{1}=20.2\;\mu m,R_{2}=19\;\mu m$) into a jumping droplet (Rj=24.7μm) under controlled condensation conditions (Tw=5°C,pv=0.87kPa).

Bioinspired geometries, such as nanocones functionally similar to cicada wings or wedge microgrooves analogous to cactus spines, optimize this balance by minimizing adhesion and controlling the coalescence diameter, typically around 20 μm to 30 μm [112,116]. By engineering these structures, surfaces sustain small droplet sizes and high surface renewal rates, resulting in persistent high heat flux and efficient condensation performance. For efficient condensation, surfaces must achieve three objectives: initiate high nucleation density, minimize adhesion for fast removal, and maintain structural stability during repeated condensation and evaporation cycles. These physical requirements including high contact angles, low hysteresis, and geometric asymmetry which directly correspond to design elements observed in natural systems [110,117].

### 6.1. Bioinspired Surfaces and Their Geometrical Translation

Many natural organisms exhibit surface morphologies that regulate water collection and droplet transport, offering physical design principles for engineered condensation surfaces. A well-known example is the Namib Desert beetle, whose elytra display a heterogeneous wettability pattern consisting of hydrophilic protrusions surrounded by hydrophobic wax-coated regions. This contrast promotes preferential droplet capture on the hydrophilic domains, followed by coalescence and gravity-assisted transport along the hydrophobic background toward the beetle’s mouth [117,118]. While this mechanism is primarily associated with fog harvesting rather than vapor condensation, the underlying principle of combining localized wetting contrast with efficient droplet removal has motivated analogous engineered designs.

Inspired by this concept, artificial surfaces with spatially patterned wettability have been explored to decouple nucleation and droplet transport during condensation. In such designs, hydrophilic regions favor droplet formation, whereas adjacent hydrophobic areas facilitate droplet mobility and shedding, improving surface renewal during condensation. This strategy reflects a broader trend in bioinspired surface engineering rather than a direct replication of a single biological system.

Beyond wettability patterning, nanoscale surface topography plays a critical role in droplet dynamics. Cicada wings are covered with dense arrays of nanoscale conical protrusions coated with a hydrophobic wax layer, rendering the surface superhydrophobic. Under condensing conditions, microdroplets forming on these nanostructures can undergo coalescence-induced jumping, enabling autonomous removal without reliance on gravity [119]. This phenomenon has directly inspired engineered nanostructured surfaces that sustain jumping-droplet condensation, leading to enhanced condensate removal and improved heat transfer performance [105]. The functional similarity between biological and artificial nanostructures lies in their ability to minimize solid–liquid adhesion and convert released surface energy into droplet kinetic energy.

Directional droplet transport is another recurring motif in biological water management. The cactus spine exemplifies this behavior, where droplets collected near the sharp tip migrate toward the base due to curvature-induced Laplace pressure gradients. This mechanism has been replicated in engineered surfaces employing conical or wedge-like geometries, which generate pressure imbalances that drive spontaneous droplet motion along prescribed directions [110]. Such geometric asymmetry enhances drainage efficiency and helps maintain active condensation regions.

Additional biological surfaces further illustrate how hierarchical structuring influences wetting and droplet mobility. Lotus leaves exhibit multiscale roughness that minimizes contact angle hysteresis and promotes droplet rolling or departure, whereas rose-petal-like textures produce high apparent contact angles but strong pinning, leading to poor droplet removal. These contrasting behaviors have been reproduced in metallic and polymeric biomimetic surfaces, demonstrating how subtle differences in roughness hierarchy can dictate condensation mode and droplet dynamics [113]. In contrast, pitcher plants have inspired liquid-infused slippery surfaces (SLIPS), where a stabilized lubricant layer suppresses pinning and enables smooth droplet sliding even on nominally flat substrates [120].

Translating these biological motifs into engineered condensation surfaces requires careful consideration of surface chemistry, structural hierarchy, and spatial organization. Rather than prescribing universal geometric optima, existing studies consistently show that reducing droplet adhesion, promoting rapid coalescence, and facilitating timely droplet removal are the dominant factors governing sustained dropwise condensation. Reviews of bioinspired condensation surfaces emphasize that successful designs integrate these principles across multiple length scales, adapting biological inspiration to the constraints of fabrication, durability, and thermal performance [121].

**Figure 15:**
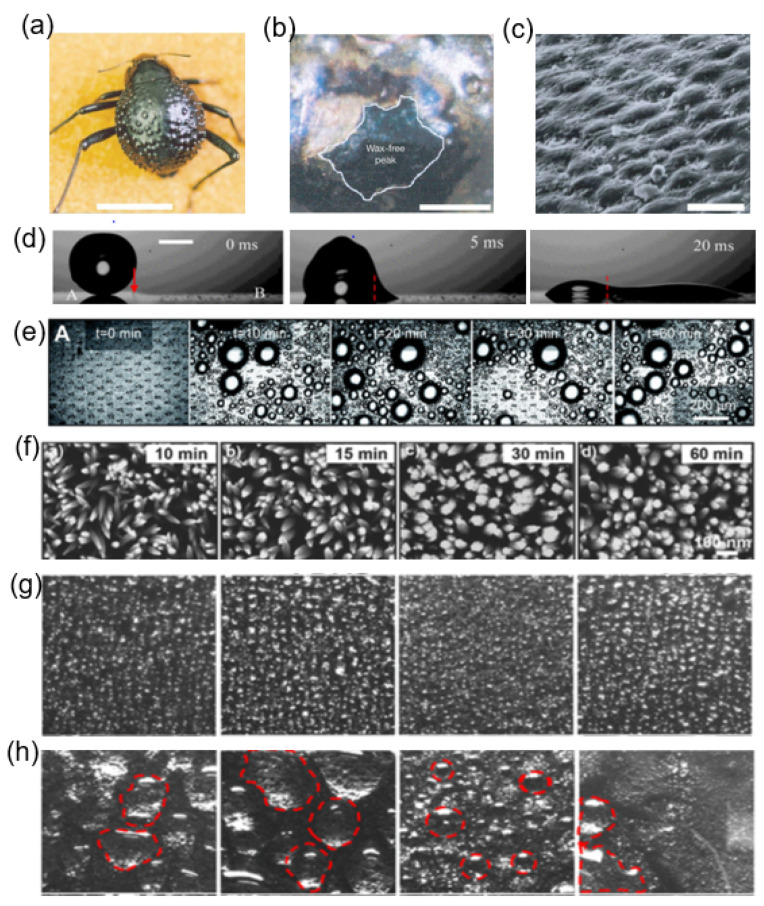
Natural fog-harvesting textures underpin the design of engineered condensation surfaces, exemplified by the Namib beetle whose elytra feature alternating hydrophilic and hydrophobic domains (**a**–**c**) that promote selective nucleation and directional transport [117]. Engineered wettability gradients similarly drive capillary-assisted droplet motion (**d**), mimicking biological pumping [110]. Time-lapse condensation on Beetle–Cactus Inspired (BCI) surfaces (**e**) demonstrates sustained nucleation and continuous droplet removal [118]. Structural control via ZnO nanoneedle arrays (**f**) tunes nucleation density for stable dropwise condensation [112], while macroscopic snapshots (**g**,**h**) show how hierarchical topography governs droplet adhesion and mobility on rose-petal- and lotus-leaf-inspired surfaces [113].

Figure 15 illustrates the bio-inspired condensation architecture. Figure 15a–c shows the Namib Desert beetle, a natural fog-harvesting organism whose elytra consist of hydrophilic peaks surrounded by hydrophobic wax-coated valleys. The hydrophilic bumps act as preferential nucleation sites, while the hydrophobic regions facilitate directional droplet transport driven by gravity or capillary forces [117]. This biological mechanism motivates engineered hybrid-wetting surfaces designed to initiate nucleation and then efficiently move droplets away from the nucleation sites. Figure 15d demonstrates this approach in an artificial setting, where a droplet placed on a surface with a controlled hydrophobic–hydrophilic boundary rapidly migrates toward the more wettable region. This motion is driven by a Laplace pressure difference, illustrating a passive pumping effect similar to beetle-inspired water collection [110]. Figure 15e,f shows the condensation performance of a Beetle–Cactus Inspired (BCI) surface. The sequential time-lapse images in (e) reveal dense nucleation, rapid coalescence, and continuous surface renewal over extended periods [118]. The SEM micrographs in (f) highlight the evolution of ZnO nanoneedles with increasing growth time, where higher aspect ratios and tighter spacing improve nucleation site density and sustain dropwise condensation [112]. Figure 15g,h compare natural and biomimetic hierarchical structures including rose-petal-like surfaces, lotus-leaf architectures, and PDMS-modified variants. On rose-petal-mimetic surfaces, droplets remain pinned and elongated due to high contact line adhesion, obstructing removal and degrading heat transfer. In contrast, lotus-leaf-inspired geometries and their PDMS-coated analogues yield nearly spherical droplets with minimal adhesion, enabling spontaneous departure and avoiding flooding. The red-circled regions in (h) emphasize continuous droplet removal zones that actively restore fresh areas for nucleation [113].

### 6.2. Demonstrated Heat Transfer Enhancement

Experimental studies consistently demonstrate that incorporating bioinspired surface geometries can enhance condensation performance by improving droplet removal and surface renewal. Miljkovic et al. [105] showed that nanostructured superhydrophobic surfaces capable of coalescence-induced droplet jumping achieved approximately a 30% enhancement in heat transfer coefficient relative to conventional dropwise condensation on smooth hydrophobic surfaces, owing to reduced droplet departure size and rapid surface refreshment. Similarly, Kim et al. [114] reported stable jumping-droplet condensation on superhydrophobic copper nanostructures, where frequent microdroplet departure accelerated surface renewal and improved condensation behavior.

Bioinspired wettability patterning has also been explored as a strategy to promote droplet mobility. Ghosh et al. [117] demonstrated that surfaces incorporating hydrophilic–hydrophobic contrasts inspired by the Namib Desert beetle enhance droplet shedding and water collection efficiency, highlighting the effectiveness of spatial wetting heterogeneity in facilitating droplet transport. More broadly, Gong et al. [110] reviewed coalescence-driven and curvature-driven droplet transport on biomimetic micro- and nanostructured surfaces, emphasizing how geometric features such as spacing and local curvature influence droplet coalescence and motion.

Advances in nanostructured metallic surfaces further illustrate the quantitative impact of bioinspired design. Wang et al. [116] fabricated densely packed copper nanocone arrays via template-free electrodeposition and reported up to a 98% enhancement in condensation heat transfer coefficient under low-temperature vapor conditions compared to flat hydrophobic copper, attributed to low droplet adhesion and sustained jumping-droplet removal. At the modeling level, Liu et al. [122] numerically investigated droplet coalescence-induced jumping on bioinspired symmetric rhombic microstructured surfaces using a lattice Boltzmann framework, showing that geometric symmetry, ridge height, and droplet size mismatch strongly influence ejection velocity, directionality, and energy conversion efficiency.

Complementary bioinspired approaches focus on reducing droplet pinning through interfacial lubrication or hierarchical roughness. Hao et al. [120] reported liquid-infused slippery surfaces inspired by pitcher plants that exhibit enhanced droplet mobility and reduced fouling tendencies, while Orejon et al. [113] demonstrated that metallic surfaces with hierarchical nano- and micro-scale features can sustain dropwise condensation over extended durations without performance degradation. Collectively, these studies show that biological concepts can be translated into engineered surface chemistries and geometries that enhance droplet nucleation control and removal efficiency. As summarized in recent reviews, bioinspired superwettability strategies provide a practical bridge between natural wetting control and thermal engineering, guiding the design of advanced surfaces for high-performance cooling, desalination, and water-harvesting technologies [112,121,123].

While bio-inspired condensation surfaces have demonstrated significant improvements in nucleation density, droplet mobility, and sustained dropwise behavior, several limitations currently restrict their practical deployment. Many nanostructured superhydrophobic coatings suffer from durability issues, including mechanical abrasion, chemical degradation, and fouling-induced loss of liquid repellency under real operating conditions. Hybrid wetting and directional-transport architectures often rely on precisely fabricated micro/nanofeatures whose manufacturing scalability and cost remain major barriers to industrial adoption. In addition, plastron stability and droplet jumping efficiency are highly sensitive to environmental variables such as steam quality, pressure fluctuations, and contaminants, leading to performance deterioration during long-term operation. To overcome these challenges, future efforts may consider focusing on more robust hierarchical textures with self-healing or lubricant-infused protection, dynamic wettability control using stimuli-responsive materials, and integrated sensing–actuation strategies to actively regulate droplet transport. Coupling biological design principles with multiphysics simulations and scalable fabrication methods will enable reliable, high-performance condensation surfaces for next-generation thermal management, desalination, and water-harvesting systems.

## 7. On Bio-Inspired Boiling Heat Transfer

Boiling is an exceptionally efficient mode of phase-change heat transfer because it couples microlayer evaporation, transient conduction, and rapid liquid–vapor exchange over microsecond timescales [123,124,125,126]. In nucleate boiling, bubbles repeatedly nucleate, grow, and depart from the surface, periodically exposing fresh liquid and renewing the solid–liquid contact area. The total heat flux is often decomposed into transient conduction and microlayer evaporation contributions, $q^{\prime \prime }=q_{\mathrm{trans}}^{\prime \prime }+q_{\mathrm{micro}}^{\prime \prime }$, a representation that is consistent with mechanistic descriptions of bubble growth and rewetting cycles [124]. Here, $q_{\mathrm{trans}}^{\prime \prime }$ accounts for unsteady conduction in the liquid and solid around a growing bubble, while $q_{\mathrm{micro}}^{\prime \prime }$ captures evaporation from the thin liquid microlayer that forms beneath the bubble footprint.

### 7.1. Classical Mechanistic Framework for Nucleate Boiling

A widely used empirical model, the Rohsenow correlation [127], embeds the influence of surface–fluid interactions in a single ‘surface factor’ $C_{sf}$: $q^{\prime \prime }=\mu_{l}h_{fg}{\left[\frac{g(\rho_{l}-\rho_{v})}{\sigma }\right]}^{1/2}\frac{{\left(\Delta T\right)}^{n}}{C_{sf}}{\left(\frac{c_{p,l}\Delta T}{h_{fg}}\right)}^{m}$, where $\mu_{l}$ is the liquid viscosity, $h_{fg}$ is the latent heat of vaporization, $\rho_{l}$ and $\rho_{v}$ are the liquid and vapor densities, σ is the surface tension, $c_{p,l}$ is the liquid specific heat, and $\Delta T=T_{w}-T_{\mathrm{sat}}$ is the wall superheat. All details of surface chemistry, roughness, and microstructure including those introduced by bioinspired designs, enter through $C_{sf}$, making it a concise parameterization of boiling enhancement [123,126,128].

The onset of nucleate boiling (ONB) is governed by heterogeneous nucleation physics. Hsu’s classical analysis [129] links the activation of a cavity of radius $r_{c}$ to a minimum wall superheat $\Delta T_{\mathrm{ONB}}\approx \frac{2\sigma T_{\mathrm{sat}}}{\rho_{v}h_{fg}r_{c}}\left(1+\frac{\cos\theta }{2}\right)$, where θ is the equilibrium contact angle. Equation shows that surfaces with re-entrant microcavities or nano-asperities effectively broaden the distribution of $r_{c}$ and can reduce $\Delta T_{\mathrm{ONB}}$ by stabilizing vapor embryos at lower superheat [130,131]. This mechanistic picture provides a direct link between cavity geometry, wettability, and the density of active nucleation sites.

Microlayer evaporation is another major contributor to boiling heat transfer. The evaporative flux from beneath a growing bubble can be scaled as $q_{\mathrm{micro}}^{\prime \prime }∼\rho_{l}h_{fg}\left(\frac{\alpha_{l}\Delta T}{\delta_{m}}\right)$, where $\alpha_{l}$ is a thermal diffusivity and $\delta_{m}$ is the microlayer thickness [124]. Surfaces that promote stable, thin microlayers over large bubble footprints enhance $q_{\mathrm{micro}}^{\prime \prime }$, while structures that accelerate microlayer drainage or rewetting can shift the relative contribution toward transient conduction.

Bubble departure characteristics set the frequency of surface renewal. Fritz’s classical force-balance model [132] gives the departure diameter $D_{d}=C{\left[\frac{\sigma }{g(\rho_{l}-\rho_{v})}\right]}^{1/2}{\left(\frac{1+\cos\theta }{2}\right)}^{-1/2}$, where *C* is an empirical constant. Hydrophobic patches or re-entrant topographies can locally modify θ, thereby tuning $D_{d}$ and the corresponding bubble departure frequency [123,126]. Surfaces that promote small, frequent bubble departure events tend to exhibit high heat transfer coefficients (HTCs) because they repeatedly disturb the thermal boundary layer and refresh the microlayer.

At sufficiently high heat flux, a boiling surface can no longer rewet between successive bubbles, leading to critical heat flux (CHF) and the formation of a stable vapor layer. Zuber’s hydrodynamic instability model [133] treats CHF as the onset of a Rayleigh–Taylor-like instability in coalesced vapor columns: $q_{\mathrm{CHF}}^{\prime \prime }=0.131\;h_{fg}\;\rho_{v}^{1/2}{\left[\sigma g(\rho_{l}-\rho_{v})\right]}^{1/4}$. In this framework, CHF is a global hydrodynamic limit independent of local surface roughness. To exceed this limit, surfaces must sustain liquid supply to the hot wall and disrupt the formation of continuous vapor chimneys [125,126,134,135].

The classical literature thus identifies four primary levers for boiling enhancement: (i) lowering $\Delta T_{\mathrm{ONB}}$ and increasing nucleation site density; (ii) tailoring microlayer geometry and lifetime; (iii) controlling bubble departure size, frequency, and coalescence; and (iv) sustaining liquid rewetting to delay CHF. Micro- and nanostructured surfaces, porous coatings, and liquid-infused layers all act on one or more of these levers [123,126,128], and modern bioinspired designs can be interpreted as hierarchical, physically motivated refinements of this mechanistic framework.

### 7.2. From Micro/Nanostructures to Multiscale Architectures

Early engineered boiling surfaces focused on introducing microstructures and porous layers that increase nucleation site density and improve liquid supply. Chu et al. [135] fabricated microstructured copper surfaces with arrays of pillars and fins. The microstructures create protected cavities at their bases and sidewalls, increasing the density of stable vapor embryos and thus reducing $\Delta T_{\mathrm{ONB}}$. At the same time, the spacing between pillars forms capillary channels that feed liquid to the hot surface while providing pathways for vapor to escape. An optimal spacing emerges from a competition between capillary-driven liquid replenishment and vapor choking.

Porous-layer coatings offer another classical route to enhancement. Liter and Kaviany [134] showed that modulated porous coatings can significantly intensify CHF by acting as capillary pumps: the interconnected pore network continuously wicks liquid to the surface and breaks up large vapor columns. Their theoretical framework links CHF to capillary pressure, permeability, and layer thickness, revealing an optimum porosity and thickness beyond which additional porous material only adds thermal resistance. These ideas anticipate later bioinspired designs that mimic vascular networks in leaves and roots.

Re-entrant and overhang structures extend this logic by stabilizing vapor embryos within geometrically protected cavities. Callenaere et al. [130] analyzed the stability of vapor bubbles in re-entrant cavities, showing that the narrow opening modifies the Laplace pressure balance and allows vapor to persist across multiple nucleation cycles. Bandyopadhyay et al. [131] experimentally demonstrated that arrays of re-entrant cavities enable early cavitation and high nucleation site densities, while their spacing and aperture geometry control bubble interaction and coalescence. These structures effectively implement Hsu’s criterion with engineered cavity size distributions and protected vapor reservoirs.

Liquid-infused and slippery surfaces, originally developed for omniphobic wetting in condensation and droplet applications, have also been extended to boiling. Wong et al. [136] introduced slippery liquid-infused porous surfaces (SLIPS) as a robust, self-healing omniphobic interface inspired by the Nepenthes pitcher plant. Yandapalli et al. [137] studied nucleate boiling on SLIPS and found that the lubricant layer reduces contact angle hysteresis and bubble pinning, enabling small, frequent bubble departure events. However, the lubricant layer also introduces a thermal resistance, so the net effect on HTC reflects a balance between improved bubble dynamics and added conduction impedance. Lou et al. [138] further examined liquid-infused porous surfaces (LIPS) and showed that by tuning lubricant viscosity and thickness, one can regulate microlayer geometry and maintain stable nucleate boiling without premature dryout. SLIPS/LIPS surfaces modulate both $\delta_{m}$ and the effective contact angle θ, thereby reshaping microlayer evaporation and bubble departure characteristics.

Comprehensive reviews by Meena et al. [126], Cho et al. [123], Sun et al. [139], Wang et al. [140], and Upot et al. [128] synthesize these developments and emphasize that high-performance boiling surfaces must simultaneously address multiple mechanisms: enhance nucleation, promote microlayer evaporation, control bubble dynamics, and maintain strong liquid supply. The finding motivates the design of hierarchical architectures in which macro-, micro-, and nanoscale structures are combined to orchestrate phase-change processes across multiple length scales.

**Figure 16:**
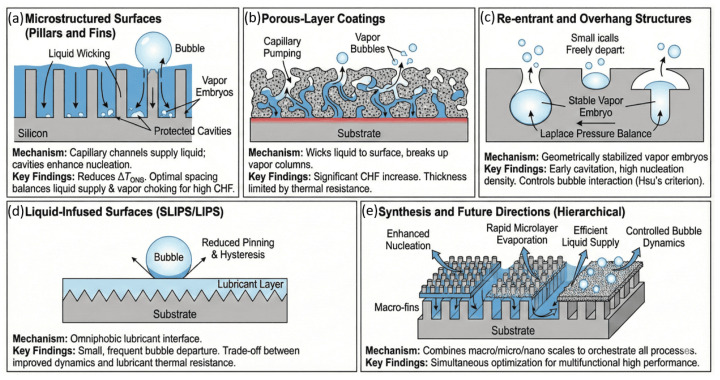
Mechanistic pathways for engineered boiling enhancement: (**a**) microstructured fins and pillars that create protected nucleation sites and promote capillary liquid transport [135]; (**b**) porous-layer coatings that wick liquid and disrupt vapor columns to increase CHF [134]; (**c**) re-entrant and overhang structures that stabilize vapor embryos and suppress bubble coalescence [130,131]; (**d**) liquid-infused surfaces that reduce bubble pinning and hysteresis, enabling frequent bubble departure [136,137,138]; and (**e**) hierarchical surfaces that integrate multi-scale features to optimize nucleation, evaporation, and liquid supply.

Engineered surface strategies for boiling enhancement are summarized in Figure 16. Microstructured pillars and fins enhance nucleation by stabilizing vapor embryos in protected cavities while supporting capillary liquid supply [135]. Porous-layer coatings intensify CHF by wicking liquid into the wall region and breaking up large vapor structures [134]. Re-entrant cavities provide geometric stabilization of vapor embryos through Laplace pressure balance, enabling early nucleation and reduced bubble coalescence [130,131]. Liquid-infused surfaces reduce contact line pinning to promote small and frequent bubble departure events [136,137,138]. Lastly, hierarchical architectures combine these multiscale effects to simultaneously improve nucleation density, microlayer evaporation, and liquid replenishment. Together, these surface innovations demonstrate how controlling liquid–vapor transport pathways can expand boiling performance beyond conventional limits.

### 7.3. Bioinspired Boiling Surfaces: Translating Natural Motifs into Design Rules

Organisms routinely manage phase change and fluid transport through hierarchical porous networks, selective wettability, and re-entrant geometries. Leaves regulate transpiration through stomata embedded in a multiscale venous network; plant roots distribute water via capillary channels; pitcher plants and other carnivorous species use lubricated overhangs to guide liquids and gas bubbles [141,142,143]. Recent boiling studies explicitly draw on these motifs to design surfaces that integrate nucleation control, liquid supply, and vapor removal.

Chen et al. [141] framed boiling enhancement as an engineered analogue of plant transpiration: liquid is drawn to the evaporating surface through capillary pathways, vapor is generated at well-defined sites, and the structure maintains a dynamic balance between water loss and supply. In this analogy, micro/nano-cavities correspond to stomata, while porous backbones and channels mimic the leaf’s xylem–phloem network. This conceptual model provides a foundation for translating biological blueprints into synthetic architectures.

A particularly clear example is the stomata-inspired boiling surface of Xu et al. [144]. They designed re-entrant cavities with pore openings and surrounding microstructures that closely resemble leaf stomata and guard cells. The pore opening serves as a spatially localized nucleation site, stabilizing a vapor embryo in a re-entrant pocket in a way consistent with the stability analysis of Callenaere et al. [130]. The adjacent microtopography and wettability gradients route liquid back into the pore after bubble departure, enabling rapid rewetting and repeated nucleation cycles at the same location. By tuning pore diameter, depth, and areal density, the surface achieves both low ONB superheats and high, spatially ordered nucleation site density, while avoiding runaway coalescence of vapor patches that would otherwise trigger CHF.

Hierarchical three-tier architectures are another powerful manifestation of bioinspired design. Song et al. [145] fabricated surfaces with macrochannels for bulk liquid and vapor transport, micropillars for localized capillarity and cavity formation, and nanostructured coatings to enhance wettability and provide a dense spectrum of nucleation sites. The macro tier functions analogously to the primary veins of a leaf, supplying fluid and venting vapor over millimeter scales; the micro tier mimics smaller veins and stomatal surroundings, distributing liquid and hosting stable bubble embryos; and the nano tier resembles the cuticle roughness, tuning local contact angles and microlayer dynamics. This multi-tier hierarchy simultaneously increases $N_{s}$, thins the microlayer, and preserves strong rewetting, resulting in large enhancements in both HTC and CHF. Ultrascalable variants of such three-tier designs [146] demonstrate that these hierarchical motifs can be realized over large areas using fabrication routes compatible with practical thermal management hardware.

Wang et al. [147] extended this model to regimes of high wall superheat, showing that properly engineered hierarchical architectures can sustain vigorous nucleate boiling without transitioning to film boiling. Their structures maintain a distributed network of active nucleation sites and capillary-fed liquid reservoirs, preventing the formation of continuous vapor layers even at large ΔT. In terms of the Zuber picture, the hierarchy effectively fragments vapor columns into smaller plumes and continually re-seeds liquid into hot spots, thereby shifting the hydrodynamic limit to higher heat fluxes.

Bioinspired design has also been applied to the bubble phase itself, through the concept of under-liquid superaerophobicity. Yu et al. [148] engineered micro/nanostructured silicon surfaces that are highly repellent to gas bubbles when submerged. Analogous to the underwater gas-repellent leaves of certain aquatic plants, these surfaces exhibit extremely low gas–solid adhesion, so that nucleated bubbles detach at small footprint diameters and high frequencies. Mechanistically, this modifies the force balance: effective contact angles and line tension are tuned so that buoyancy and flow-induced forces overcome surface anchoring at smaller $D_{d}$. The result is rapid surface renewal, reduced risk of vapor blanket formation, and enhanced nucleate boiling performance.

Smart slippery and liquid-infused designs further extend biomimetic concepts to boiling. The original SLIPS concept mimics the lubricated peristome of the Nepenthes pitcher plant [136], where a thin liquid film anchored in a nano/microstructured solid provides nearly frictionless, omniphobic transport. In the boiling context, Yandapalli et al. [137] and Lou et al. [138] demonstrated that such slippery interfaces enable easy bubble nucleation and detachment, low hysteresis, and self-cleaning behavior, albeit with added thermal resistance and challenges related to lubricant longevity. Recently, Shi et al. [149] designed bioinspired smart slippery surfaces for controlled bubble manipulation in microscale boiling environments. By spatially patterning lubricant retention structures and wettability, they created analogues of directional peristome ribs or anisotropic leaf cuticles that steer bubbles along preferred paths, prevent vapor accumulation in critical regions, and potentially couple boiling to sensing or actuation. These systems transform boiling from a passive response to heat input into a programmable, spatially organized phase-change process.

Bioinspired coatings also play an important role in flow boiling. Mohammadilooey et al. [150] investigated the effect of porous bio-coatings on flow boiling of HFE-7000. Their surfaces, inspired by the porous, compliant architectures of biological tissues, provide both additional nucleation sites and capillary wicks that sustain liquid supply under high shear. By tuning coating porosity and thickness, they identified regimes where enhancements in nucleation and CHF outweigh added conduction resistance, echoing the optimization principles of porous-layer coatings [134] but realized with bio-derived or bioinspired materials.

Beyond individual surface designs, several recent reviews synthesize how bioinspired topologies organize fluid flow and phase change across scales. Zhang et al. [142] evaluated bioinspired topological surfaces for mitigating water, thermal, and energy crises, highlighting how motifs such as cactus spines, beetle elytra, leaf veins, and shark skin can be abstracted into design rules for capillary pumping, directional transport, and turbulence control. Wang et al. [151] reviewed bioinspired slippery surfaces for manipulating liquids from tiny droplets to bulk flows, emphasizing the multifunctionality anti-fouling, anti-icing, drag reduction, and phase-change enhancement that arises from combining lubricant layers with hierarchical textures. Upot et al. [128] and Yang et al. [152] provide a unified perspective on micro- and nanoengineered surfaces for both boiling and condensation, showing that the same structural elements (re-entrant cavities, hierarchical roughness, lubricant-infused pores) can be tuned to support either vigorous bubble nucleation or efficient droplet shedding depending on the thermodynamic driving force.

**Figure 17:**
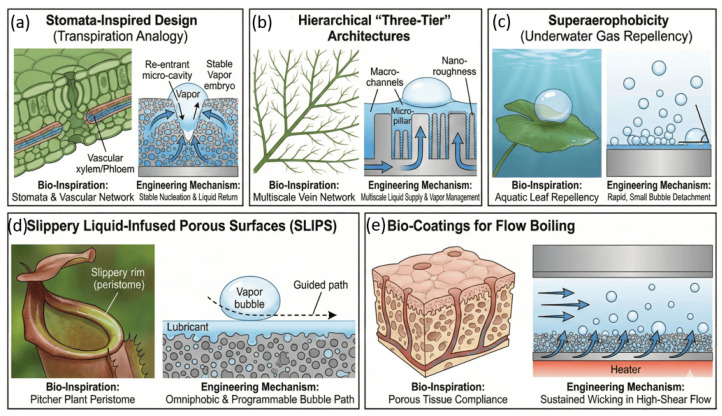
Bio-inspired boiling surface architectures and their mechanistic analogues in natural systems: (**a**) stomata-inspired surfaces with re-entrant cavities and capillary channels that stabilize vapor embryos and sustain liquid return [141,144]; (**b**) hierarchical three-tier architectures that integrate macro-, micro-, and nano-features to enhance nucleation density, microlayer evaporation, and fluid transport [145,146,147]; (**c**) superaerophobic leaf-inspired micro/nanostructures that suppress gas adhesion and promote rapid bubble detachment [148]; (**d**) SLIPS coatings modeled after pitcher-plant peristomes to reduce bubble pinning and enable controlled bubble motion [136,137,138,149]; and (**e**) bio-inspired flow-boiling coatings that mimic porous tissues to provide sustained liquid wicking and improved robustness under high-shear conditions [150].

Figure 17 illustrates major categories of bioinspired boiling surfaces that connect biological fluid-transport strategies to engineered phase-change enhancement. Stomata-inspired re-entrant cavities guide liquid supply and stabilize vapor embryos in analogy to leaf transpiration networks [141,144]. Hierarchical three-tier architectures integrate macrochannels, micropillars, and nanoscale roughness to simultaneously increase nucleation site density and maintain persistent liquid replenishment [145,146,147]. Underwater superaerophobic surfaces mimic aquatic foliage to minimize bubble adhesion and accelerate bubble departure [148]. Slippery liquid-infused porous surfaces emulate the omniphobic transport of pitcher plants, enabling guided bubble motion and reduced contact-line hysteresis [136,137,138,149]. Finally, compliant porous tissue-inspired coatings sustain wicking under high-shear flow conditions and promote robust nucleate boiling during flow boiling [150]. Collectively, these biologically informed motifs demonstrate how nature-derived design principles address the coupled requirements of nucleation control, microlayer regulation, liquid resupply, and vapor evacuation in extreme boiling environments.

Overall, classical boiling theory identifies the central mechanisms controlling nucleation, microlayer evaporation, bubble dynamics, and CHF [126]. Micro- and nanostructured surfaces, porous coatings, and liquid-infused layers operationalize these mechanisms by tailoring cavity populations, capillary networks, and interfacial mobility [123,137,138,139,140]. Bioinspired boiling architectures go one step further: they draw explicit analogies to leaves, stomata, vascular networks, and slippery plant peristomes to design hierarchical, multifunctional surfaces that simultaneously enhance nucleation, accelerate bubble departure, and sustain liquid supply [142,143,147,148,149,150,151]. These strategies provide a physically grounded pathway to move beyond empirical enhancement toward rational, bioinspired surface design for extreme boiling heat transfer. Table 3 summarizes the bioinspired boiling architecture and the mechanistic enhancement.

Despite substantial advances in bioinspired boiling architectures, several critical challenges limit their practical deployment. First, many micro/nanostructured surfaces experience durability limitations under high heat flux, fouling, and chemical corrosion, leading to loss of wettability or capillary functionality during long-term operation. Second, hierarchical and re-entrant geometries often rely on complex fabrication techniques that limit scalability and compatibility with large-area industrial heat exchangers. Lubricant-infused slippery surfaces introduce additional concerns related to lubricant depletion, thermal resistance, and stability under shear-driven flows. Optimized boiling performance depends on a delicate balance among nucleation density, liquid replenishment, and vapor escape, which can shift under transient thermal loads and coolant contamination. To overcome these barriers, future work may emphasize scalable manufacturing methods, mechanically robust surface chemistries, and self-replenishing or self-healing textures inspired by biological regeneration. Integrating real-time sensing and active fluid control into these biomimetic designs will also enable adaptive phase-change surfaces that maintain high HTC and delayed CHF across dynamic operating conditions. Such advancements will accelerate the transition of bioinspired boiling technology toward reliable thermal management in power electronics, desalination, and next-generation energy systems.

## 8. Conclusions and Future Work

This review has synthesized recent advances in bio-inspired strategies for flow control, fluid–structure interaction, and thermal transport through a mechanism-driven perspective. Rather than cataloging biological analogues, the article emphasized the underlying physical processes such as unsteady vortex dynamics, compliance-enabled flow tuning, capillary-mediated transport, and interfacial regulation that enable multifunctional performance across aerodynamic, hydrodynamic, and thermal systems. By organizing bio-inspired designs around shared transport mechanisms and nondimensional scaling principles, the review provides a unified framework for evaluating when biological inspiration yields fundamentally new transport pathways versus incremental optimization within classical fluid-mechanical limits. The analysis demonstrates that bio-inspired flow control strategies effectively manipulate separation, wake topology, and near-wall momentum transport, while bio-inspired fluid–structure interaction systems exploit compliance and unsteady kinematics to enhance propulsion, mixing, and energy harvesting. In parallel, bio-inspired phase-change heat-transfer surfaces leverage hierarchical geometry and wettability control to regulate nucleation, liquid replenishment, and droplet or bubble removal. Together, these domains reveal strong conceptual linkages driven by multiscale geometry, vortex–surface interactions, and adaptive structural response.

Despite this progress, the review highlights several open research questions that must be addressed to advance bio-inspired concepts from laboratory demonstrations to robust engineering technologies. A critical unresolved issue is how tightly coupled fluid–structure–rheology interactions can be modeled and exploited, particularly in biological and engineered systems operating in non-Newtonian or viscoelastic environments. Future work can focus on determining how elastic stress relaxation, shear-dependent viscosity, and interfacial viscoelasticity modify wake dynamics, force generation, and transport efficiency across relevant Reynolds and Deborah number regimes. Another key research direction is the integration of structural compliance into phase-change heat-transfer surfaces. While rigid textured surfaces dominate current boiling and condensation studies, biological systems suggest that dynamically deformable microstructures could actively regulate bubble nucleation, coalescence, and detachment. Fundamental questions remain regarding how compliant surface elements interact with transient vapor–liquid interfaces, how such interactions influence critical heat flux and surface longevity, and how these effects scale across length and time scales. The review also identifies a need for unified, rheology-aware nondimensional frameworks that extend beyond classical Reynolds, Weber, and Capillary numbers. Incorporating parameters such as the Weissenberg and Deborah numbers into bio-inspired design maps may enable systematic comparison of Newtonian and non-Newtonian operating regimes and guide the selection of optimal actuation frequencies, material stiffness, and surface architectures. Finally, future research may explore scalability, durability, and system-level integration. Open questions include how bio-inspired micro- and mesostructures can be manufactured reliably at engineering scales, how multifunctional performance can be preserved under realistic environmental loading and fouling, and how data-driven control and optimization methods can be coupled with high-fidelity multiphysics models to enable adaptive, resilient operation.

By framing these challenges as concrete research questions, this review aims to guide future investigations toward mechanistically grounded, scalable bio-inspired designs. Continued progress will depend on integrating fluid mechanics, structural dynamics, rheology, materials science, and data-driven modeling to translate the adaptive strategies of biological systems into deployable thermal–fluid technologies.

## Figures and Tables

**Table 2:** Summary of fluid-mechanical mechanisms and performance improvements in bio-inspired wave-energy systems.

Concept	Bio Source	Fluid-Mechanical Mechanism	Outcomes	Ref.
Folding-wing WEC	Flying fish fins	Resonant flapping enhances periodic lift–drag exchange and strengthens wave–structure coupling.	∼30% increase in capture-width ratio at a 1.4 s wave period.	Chen et al. [45]
Scallop-shell flap	Scallop valves	Outward curvature increases pressure differential and reduces reverse-flow losses.	∼30% higher capture factor relative to a rectangular flap.	Wang and Liu [46]
Electric-eel generator	Electric eel	Vortex-induced vibration (VIV) amplifies transverse oscillations in weak currents.	Stable operation at ∼0.4 m/s, whereas conventional WECs require >1 m/s.	Zhou et al. [47]
Dolphin-like converter	Dolphin body	Streamlined geometry suppresses adverse vortex shedding and reduces hydrodynamic damping.	Hydraulic efficiency +8–10%; electromagnetic efficiency +5–10%.	Patil et al. [48]
Jellyfish bj-TENG	Jellyfish bell	Compliant bell structure mitigates VIV and stabilizes surface-pressure fluctuations.	143 V and 11.8 mA/$m^{2}$ at 0.75 Hz.	Chen et al. [49]
Segmented sea-snake TENG	Sea snake	Spring-linked, multi-segment body conforms to wave curvature and lowers hydrodynamic resistance.	3.5 W/$m^{3}$; output voltage increased by 350% via tapered springs.	Zhang et al. [50]
Kelp-like TENG	Kelp blades	Slender, flexible blades reduce separation while supporting large-amplitude oscillations.	∼260 V and 0.7 μA/${\mathrm{cm}}^{2}$ at 1 Hz.	Wang et al. [51]
Smooth-shark dual-raft WEC	Shark body contour	Semi-elliptical body form modulates vortex distribution and promotes asymmetric excitation.	Maximum capture factor of 1.93 at a 3 s wave period.	Li et al. [57]
Venus-flytrap PEH	Venus flytrap trap	Bistability combined with VIV induces snap-through motion for high-power output bursts.	181 V and 1.18 mW vs. 0.143 mW for conventional designs.	Qian et al. [53]
Fish-shaped BF-TEHG	Fish swimming motion	Two-stage swinging motion converts fluctuating lift and drag into electrical energy.	Efficient operation at 0.24 m/s.	Gao et al. [54]
Honeycomb hybrid How-NG	Honeycomb cells	Resonance tuning combined with dual-mode energy conversion enhances uptake.	Improved performance under irregular wave conditions.	Feng et al. [55]
Dragonfly BDFH-TEHG	Dragonfly wings	Phase-lagged tandem hydrofoils exploit constructive oscillations for improved harvesting.	Start-up at 0.21 m/s; stable operation up to 0.61 m/s.	Dong et al. [56]
Soft-fin SF-TEG	Fish fin	Large, compliant deformation amplifies hydrodynamic loading and harvested power.	High efficiency at low flow velocities.	Zhang et al. [52]

**Table 3:** Summary of bioinspired boiling architectures and their mechanistic enhancements.

Bioinspired Concept	Mechanistic Strategy	Boiling Performance Benefits	Representative Studies
Transpiration-analogue vascular networks	Capillary-driven liquid supply via porous channels and micro/nanocavities.	Sustained rewetting; stabilized vapor embryos; delayed CHF.	Chen et al. [141], Zhang et al. [142]
Stomata-inspired re-entrant cavities	Protected nucleation sites with wettability gradients for pore re-seeding.	Low ONB superheat; ordered nucleation; reduced vapor-patch coalescence.	Callenaere et al. [130], Xu et al. [144]
Three-tier hierarchical architectures	Macrochannels for transport; micropillars for capillarity; nanostructures for activation density.	Higher HTC and CHF; dense active sites; microlayer thinning.	Song et al. [145], Li et al. [146]
High-superheat stability surfaces	Distributed nucleation with capillary reservoirs preventing vapor blanket formation.	Boiling sustained at high ΔT; higher hydrodynamic limit.	Wang et al. [147]
Under-liquid superaerophobic surfaces	Low gas–solid adhesion enabling rapid, low-footprint bubble detachment.	Fast surface renewal; reduced dry area; improved heat transfer.	Yu et al. [148]
SLIPS / smart SLIPS	Lubricant-infused textures mimicking pitcher-plant peristomes.	Low hysteresis; guided bubble motion; self-cleaning behavior.	Wong et al. [136], Yandapalli et al. [137], Lou et al. [138], Shi et al. [149]
Bioinspired porous coatings	Compliant porous layers distributing liquid and anchoring vapor embryos.	Enhanced CHF even in shear flows; improved nucleation density.	Liter and Kaviany [134], Mohammadilooey et al. [150]
Topological transport surfaces	Directional microtextures emulating cactus spines, leaf venation, and shark skin.	Strong pumping; controlled bubble pathways; minimized flooding.	Zhang et al. [142], Wang et al. [143,151]

## Data Availability

No new data were created or analyzed in this study. Data sharing is not applicable to this article.
